# Consecutive fecal microbiota transplantation for metabolic dysfunction-associated steatotic liver disease: a randomized controlled trial

**DOI:** 10.1080/19490976.2025.2541035

**Published:** 2025-08-04

**Authors:** Bas Groenewegen, Merel M. Ruissen, Emily Crossette, Rajita Menon, Amanda L. Prince, Jason M. Norman, Bart E. P. B. Ballieux, Hildo J. Lamb, Elisabeth M. Terveer, Josbert J. Keller, Maarten E. Tushuizen

**Affiliations:** a Netherlands Donor Feces Bank, Leiden University Center of Infectious Diseases (LUCID) Medical Microbiology and Infection Prevention, Leiden University Medical Center (LUMC), Leiden, The Netherlands; b Department of Gastroenterology and Hepatology, LUMC, Leiden, The Netherlands; c Department of Internal Medicine, LUMC, Leiden, The Netherlands; d Vedanta Biosciences Inc, Cambridge, MA, USA; e Department of Clinical Chemistry, LUMC, Leiden, The Netherlands; f Department of Radiology, LUMC, Leiden, The Netherlands; g Department of Gastroenterology and Hepatology, Haaglanden Medical Center, The Hague, The Netherlands

**Keywords:** Fecal microbiota transplantation (FMT), metabolic dysfunction-associated steatotic liver disease (MASLD), NAFLD, MRI-PDFF, hepatic steatosis, glucose tolerance, insulin resistance, gut microbiota, microbiota diversity, microbiota engraftment

## Abstract

The gut microbiota is increasingly considered a contributory factor in metabolic dysfunction-associated steatotic liver disease (MASLD). This double-blind RCT evaluated the effect of three consecutive fecal microbiota transplantations (FMT) on hepatic steatosis in MASLD. Twenty patients with MASLD were randomized (1:1) to receive allogeneic or autologous FMTs at weeks 0, 3, and 6, with follow-up through week 12. FMT material was derived from two donors. We assessed changes in hepatic steatosis (magnetic resonance imaging-derived proton density fat fraction (MRI-PDFF)), glucose tolerance (oral glucose tolerance test), liver biochemistry, and gut microbiota composition/engraftment. Change in MRI-PDFF from baseline to week 12 was not

significantly different between groups (*p* = 0.50). Liver biochemistry and glucose tolerance also showed no significant overall changes. Patients’ stool microbiota exhibited high baseline alpha diversity and similar composition across treatment groups, diverging by week 12 (*p* = 0.02). Two microbial taxa belonging to the families *Gastranaerophilaceae* and *Rikenellaceae* were associated with triglyceride levels after FMT. No further microbiota signatures were associated with FMT-treatment or response. Donor microbiota engraftment appeared donor-specific, but not treatment- or response-specific. In conclusion, FMT did not significantly affect hepatic steatosis, glucose tolerance, liver biochemistry, or gut microbiota signatures. Future studies should consider including patients with low microbiota diversity. Dutch Trial Register: NL-OMON48776; Central Committee on Research Involving Human Subjects: NL66705.058.18; Clinicaltrials.gov: NCT04465032.

## Introduction

Metabolic dysfunction-associated steatotic liver disease (MASLD) is a significant global health challenge, affecting approximately 30% of the general population,^1,2^ rising to 55% in patients with type 2 diabetes mellitus,^3^ and 71–92% those who are obese to morbidly obese.^4,5^ It encompasses a range of phenotypes, from simple steatosis, characterized by lipid accumulation in >5% of hepatocytes, to metabolic dysfunction-associated steatohepatitis (MASH), adding hepatic inflammation and/or fibrosis, and MASH-related cirrhosis and hepatocellular carcinoma (HCC).^6–8^

Obesity and insulin resistance are strongly associated with MASLD, mostly through increased delivery of free fatty acids to the liver and increased hepatic lipogenesis associated with hyperglycemia and hyperinsulinemia.^9,10^ Other contributory factors are divided into those with an established association with MASLD, including genetic factors (e.g. polymorphism of patatine-like phospholipase domain-containing protein 3 gene),^11^ dietary factors (e.g. fructose),^12,13^ and adipokines,^10^ as well as those with a potential association needing validation, including dysbiosis of the gut microbiota.

The implication of the gut microbiota in MASLD is increasingly recognized. Via the “gut-liver axis,” the liver may be exposed to gut microbiota-derived products, including toxins, enzymes, bile acids, and short-chain fatty acids (SCFAs).^14,15^ Additionally, gut microbial ethanol production may induce MASLD, with suspected involvement from *Lactobacillus, Streptococcus* and *Klebsiella* species.^15,16^ Furthermore, elevated gut ethanol may negatively affect *Akkermansia muciniphila*, a gut bacterium inversely associated with metabolic syndrome.^17,18^ However, literature on a specific MASLD-gut microbiota-signature remains inconsistent.^19^

Modulation of the gut microbiota and alterations in gut barrier integrity may affect the nature and quantity of microbiota-derived products reaching the liver, and their downstream effects on liver lipid metabolism and inflammation.^14,20,21^ In humans, fecal microbiota transplantation (FMT) from lean to obese individuals has been suggested to improve hepatic insulin sensitivity in individuals with metabolic syndrome, with preliminary clinical evidence indicating attenuation of MASLD, although results of published reports are conflicting.^22–25^ Heterogeneity in these previous studies warrants controlled studies into the effects of microbiota modulation on MASLD and the patient microbiota, to unlock its therapeutic potential.

In this randomized controlled trial, patients with MASLD were recruited to measure the effects of three consecutive allogeneic versus autologous FMT treatments on liver fat accumulation, measured by magnetic resonance imaging-derived proton density fat fraction (MRI-PDFF), and secondary clinical and metabolic MASLD-related parameters. Additionally, clinical response to treatment was associated with changes in the patient gut microbiota and engraftment of the donor microbiota.

## Methods

### Patients

Twenty patients, males and postmenopausal females aged 18–70 y, with a BMI > 27 kg/m^2^ and hepatic steatosis were recruited from the outpatient clinics of the Department of Gastroenterology and Hepatology of the Leiden University Medical Center (LUMC) and affiliated hospitals. Hepatic steatosis was defined by moderate to severe liver hyperechogenicity on abdominal ultrasound and/or FibroScan with controlled attenuation parameter >278 dB/m, according to clinical practice guidelines.^26^ Exclusion criteria included excessive ethanol consumption (>2 units/d); recent antibiotic use (<3 months); use of medications affecting microbiota or recent changes in dosages (<3 months); recent significant weight change (>5%); cardiovascular co-morbidities; previous use of glucocorticosteroids and hormonal substitution/therapy (Supplement 1). Written informed consent was obtained from patients for their participation in the study and donors for use of donor fecal samples. This was approved by the Medical Ethics Committee Leiden, The Hague, Delft (P18.201, P15.145). This study conforms to the 1975 Declaration of Helsinki.

### Study design and outcome measures

**Figure 1.**
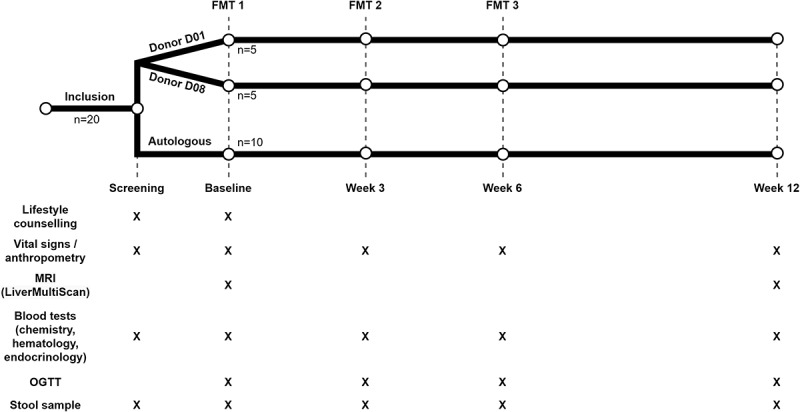
Study design. At inclusion, 20 patients were randomized into the allogeneic FMT and autologous FMT groups. After a screening visit, patients received three FMT infusions at baseline, week three, six, and twelve. Liver fat was measured at week zero and 12. X indicates when a measurement was performed or a sample was collected. FMT: fecal microbiota transplantation; MRI: magnetic resonance imaging; OGTT: oral glucose tolerance test.

This study was conducted as a single center randomized placebo-controlled trial (Figure 1). Patients were randomized to receive either allogeneic (*n* = 10) or autologous (*n* = 10) FMT at baseline, week three, and week six. For allogeneic FMT, two screened, healthy, lean donors (D01 and D08) with a BMI between 20 and 25 kg/m^2^ were used; five patients received FMT from D01 and five from D08. Sample size calculations are provided in Supplement 1. The primary outcome was the change in liver fat accumulation from baseline to week 12, measured by MRI-PDFF, following consecutive allogeneic versus autologous FMT. MRI-PDFF was quantified following the LiverMultiScan (Perspectum Ltd, Oxford, UK) protocol for image acquisition,^27^ as described by Schaapman et al.,^28^ and Shumbayonda et al.^29^ Secondary outcome parameters were derived from anthropometric data, and blood and stool samples collected at each study visit (Table 1). Additional metabolic parameters were obtained by seven-point oral glucose tolerance test (OGTT) at each study visit. During the OGTT, patients consumed 75 g of glucose in 100 ml water after an overnight fast.^30^ Blood samples were collected at 0, 10, 20, 30, 60, 90 and 120 min to determine areas under the curve (AUC) and incremental areas under the curve (iAUC) for glucose, insulin, c-peptide, glucose-dependent insulinotropic peptide (GIP), and glucagon-like peptide-1 (GLP-1). AUCs and iAUCs were calculated using the trapezoidal method. The Homeostasis Model Assessment of Insulin Resistance (HOMA-IR) and pancreatic β-cell function (HOMA-B) were calculated from baseline fasting insulin and glucose levels.^31^

**Table 1.** Baseline characteristics of included patients.

	Autologous FMT (*n* = 10, mean, [SD])	Allogeneic FMT (*n* = 10, mean, [SD])	Statistics (*p*-value, [95% CI])
Sex female	n = 6	n = 6	–
Age (years)	63.20, [5.12]	62.30, [4.88]	0.69, [−3.80–5.60]
BMI (kg/m2)	33.96, [6.41]	34.01, [6.77]	0.99, [−6.25–6.15]
Abdominal circumference (cm)	116.50, [16.37]	113.50, [13.02]	0.66, [−10.90–16.90]
Systolic blood pressure (mmHg)	138.85, [16.86]	140.55, [12.02]	0.80, [−15.46–12.06]
Diastolic blood pressure (mmHg)	84.30, [3.99]	82.35, [3.44]	0.26, [−1.55–5.45]
Albumin (g/l)	45.00, [3.06]	44.90, [2.08]	0.93, [−2.36–2.56]
ASAT (U/l)	46.22, [41.32]	30.33, [12.51]	0.29, [−14.62–46.39]
ALAT (U/l)	65.30, [71.72]	49.20, [20.05]	0.50, [−33.37–65.57]
ALP (U/l)	78.90, [19.33]	82.00, [26.34]	0.77, [−24.81–18.61]
gGT (U/l)	44.50, [53.50]	28.70, [10.25]	0.37, [−20.39–51.99]
Total bilirubin (µmol/l)	9.60, [6.93]	10.50, [4.65]	0.74, [−6.44–4.64]
Cholesterol (mmol/l)	5.90, [1.71]	5.15, [0.80]	0.23, [−0.51–2.00]
LDL (mmol/l)	3.68, [1.43]	3.07, [0.73]	0.24, [−0.46–1.68]
HDL (mmol/l)	1.39, [0.56]	1.50, [0.34]	0.61, [−0.54–0.33]
Cholesterol:HDL ratio	4.43, [0.83]	3.59, [0.97]	0.05, [−0.01–1.69]
C-reactive protein (mg/l)	3.45, [3.47]	2.84, [2.06]	0.64, [−2.07–3.29]
Triglycerides (mmol/l)	1.81, [0.72]	1.27, [0.65]	0.10, [−0.11–1.19]
MRI-PDFF (%)	15.74, [8.39]	18.6, [9.07]	0.47, [−11.07–5.35]
Fasting glucose (mmol/l)	7.40, [1.40]	6.20, [0.93]	0.06, [−0.04–2.44]
Fasting insulin (mIU/l)	30.76, [20.28]	21.92, [7.23]	0.22, [−6.04–23.72]
Fasting c-peptide (nmol/l)	1.63, [0.57]	1.24, [0.23]	0.07, [−0.04–0.82]
Fasting GIP (pmol/l)	8.22, [4.20]	5.23, [2.81]	0.08, [−0.43–6.42]
Fasting GLP-1 (pmol/l)	8.52, [4.63]	5.84, [4.38]	0.21, [−1.69–7.04]
HOMA-IR	10.44, [7.29]	6.18, [2.55]	0.11, [−1.07–9.60]
HOMA-B	169.92, [149.34]	167.93, [52.98]	0.97, [−107.48–111.46]

FMT: fecal microbiota transplantation; BMI: body mass index; ASAT: aspartate aminotransferase; ALAT: alanine aminotransferase; ALP: alkaline phosphatase; gGT: gamma-glutamyltransferase; LDL: low-density lipoprotein; HDL: high-density lipoprotein; MRI-PDFF: magnetic resonance imaging-derived proton density fat fraction; GIP: Gastric inhibitory polypeptide; GLP-1: Glucagon-like peptide-1; HOMA-IR/B: Homeostasis Model Assessment of Insulin Resistance/β-cell function.

A post-hoc clinical response category was defined by a > 5% reduction in MRI-PDFF, HOMA-IR, and serum triglycerides at week 12, aiming to reflect the metabolic interplay between insulin resistance, elevated free fatty acids, and increased hepatic and serum triglycerides, as summarized by Ruissen et al. (2020).^8^ Due to missing HOMA-IR values, combined decrease in MRI-PDFF and triglycerides was used in 6/20 cases. Stool samples from both patients and donors were used to determine microbiota composition and engraftment.

### FMT preparation

FMT material was processed and provided by the Netherlands Donor Feces Bank (NDFB, Leiden, the Netherlands), which adheres to standardized procedures for the collection, screening, preparation and storage of donor fecal suspensions.^32,33^ In short, each FMT treatment contained 60 g of feces, processed into a 198 ml feces suspension with 10% added glycerol, stored at −80°C until the day of FMT. FMT was administered directly into the duodenum via gastroduodenal endoscopy by an experienced gastroenterologist.

### Metagenomic sequencing of stool samples

DNA extraction was performed using the Qiagen DNeasy 96 PowerSoil Pro QIAcube HT extraction kit and protocol. Extracted DNA was assessed for DNA concentration, A260/A230, and A260/A280 using the DropSense platform. Samples were further quantified using broad-range Picogreen. Library preparation was performed using the KAPA HyperPlus library preparation protocol. Library-prepared DNA quality and quantity was assessed using Picogreen. Sequencing was performed using the Illumina NovaSeq instrument and protocol. Four control samples were included on each 96-well sequencing plate. Raw read classification was performed using the Kraken 2 platform,^34^ using the Genome Taxonomy Database (GTDB), release R202, followed by Bracken for abundance estimation.^35^

### Metagenomics data analysis

Metagenomics data was processed and analyzed mainly using the R software packages: phyloseq (v1.50.0), tidyverse (v2.0.0), and microbiome (v1.28.0). For analyses of microbiota diversity, engraftment and similarity, raw data was filtered to a relative taxon abundance ≥0.01%. Microbial alpha diversity was determined by calculating the Shannon index at the species level, and read counts of each metagenome were rarefied to the lowest read count of 2,899,480. Microbial beta-diversity was assessed using Bray-Curtis dissimilarity at the species level. For differential abundance testing, raw data was filtered to a mean relative taxon abundance ≥0.01%. Species engraftment fraction (SEF) and microbiota similarity were calculated as described by van Lingen et al.,^36^ with minor adaptations (Supplement 1). In short, SEF measures the proportion of donor species present in patients post-FMT, excluding those already present in patients pre-FMT. Microbiota similarity was assessed using a Bray-Curtis dissimilarity-based approach, converted to similarity scores (1 – dissimilarity).

### Statistical analysis

Statistical analyses were performed using R (v4.4.2) software. Continuous data were presented as mean and standard deviation (SD). Relative difference in MRI-PDFF between baseline and week 12 across intervention groups was assessed by independent samples t-test. Linear regression was applied for assessment of donor-specific effects. For secondary outcomes, including clinical, metabolic, and gut microbiota measures, linear mixed effects models (LMMs) were applied to assess longitudinal changes between intervention groups, using packages lme4 (v1.1–35.5) and LmerTest (v3.1–3) (Supplement 1). LMMs for assessment of longitudinal differential abundance were performed using LinDA (v0.2.0)^37^ on raw taxonomic data. Resulting taxa lists were pruned based on mean relative taxon abundance, and *p-*values were adjusted using the Benjamini & Hochberg procedure. For gut microbiota parameters, additional LMMs were performed to evaluate longitudinal changes between response groups and assess associations with MRI-PDFF, HOMA-IR and serum triglycerides between treatment groups and irrespective of treatment (Supplement 1). Differences in microbial beta-diversity were tested using permutational multivariate analysis of variance (PERMANOVA) with 999 permutations and beta dispersion testing (vegan package v2.6–8). Wilcoxon and Kruskal–Wallis tests were performed to compare relative abundances of specific, potentially MASLD-associated organisms between donors or treatment groups, respectively. Missing data in OGTT time-series were addressed using multiple imputation (Supplement 1).

## Results

### The effect of consecutive FMT on liver fat content, anthropometric parameters, inflammation, and liver function

**Figure 2.**
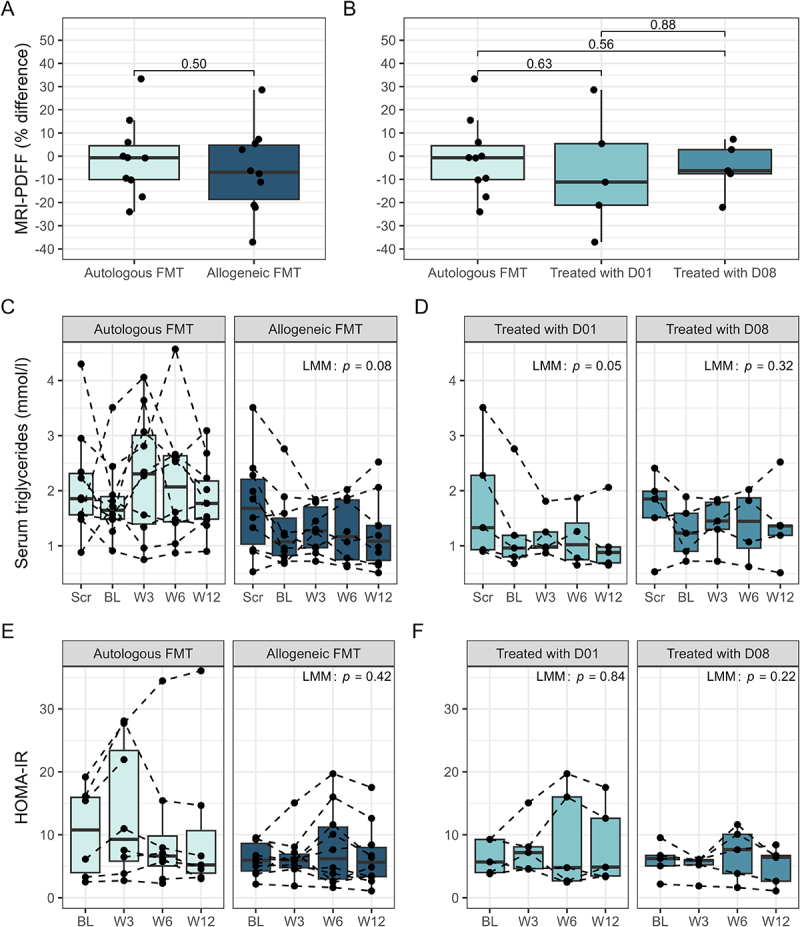
MASLD-associated clinical parameters over time. A, B: comparison of patient Δliver fat (%) (baseline to week 12), measured by magnetic resonance imaging-derived proton density fat fraction (MRI-PDFF), between intervention groups. C, D: serum triglycerides over time, separated by intervention group. E, F: homeostatic model assessment for insulin resistance (HOMA-IR) over time, separated by intervention group. Panels A and B show *p*-values from independent samples t-test and linear regression, respectively. Panels C-F show *p*-values from linear mixed effect models (LMM), comparing intervention groups over time (baseline to week 12). FMT: fecal microbiota transplantation; D01: donor one; D08: donor eight.

Analysis of the change in liver fat content from baseline to week 12 showed no significant differences between treatment groups receiving consecutive allogeneic or autologous FMT (relative difference −6.13%, SD 18.44%, versus −0.79%, SD 16.54%, 95% CI [−11.14–21.80], *p* = 0.50) (Figure 2A). Additionally, no donor-specific effects were observed (relative difference 0.79%, SD 16.54%, 95% CI [−12.81–11.22] (autologous FMT), versus −6.29%, SD 25.20%, 95% CI [−27.10–14.52] (D01), versus −4.38% SD 11.31%, 95% CI [−25.19–16.43] (D08), *p* = 0.79) (Figure 2B). A nonsignificant trend toward a decrease in triglycerides (*p* = 0.08) was observed in patients treated with allogeneic FMT, primarily attributed to treatment with D01 (*p* = 0.05) (Figure 2C,D, Table S1). No significant effects were observed upon the analysis of other parameters regarding anthropometry, inflammation, and liver function (Table S1, Figures S1–5).

### The effect of consecutive FMT on metabolic parameters

AUCs and iAUCs for glucose, insulin, c-peptide, GIP, and GLP-1, derived from the OGTT were not significantly different over time between the allogeneic and autologous FMT group, with no significant donor-specific effect (Table S2, Figures S6, 7). Additionally, no difference in HOMA-IR was observed between the treatment groups (estimate −1.37, 95% CI [−4.50–1.76], *p* = 0.42) or among different FMT donors (estimate −0.37, 95% CI [−3.73–2.98], *p* = 0.84 (D01), versus estimate −2.43, 95% CI [−5.84–0.97], *p* = 0.22 (D08)) (Figure 2E,F). Similar results were observed for HOMA-B (Table S1, Figure S6A, B).

### Clinical and metabolic response

Patients matching the post-hoc clinical response category were found in both treatment groups (allogeneic FMT: 4/10, autologous FMT: 3/10) (Table S3). Response irrespective of treatment group was associated with a decrease in c-peptide AUCs (*p* = 0.02) (Table S4). Specific response to allogeneic FMT was associated with decreased insulin AUCs (*p* = 0.03) and a trend toward reduced c-peptide AUCs over time (*p* = 0.05) (Table S4).

### The effect of consecutive FMT on patient gut microbiota composition

**Figure 3.**
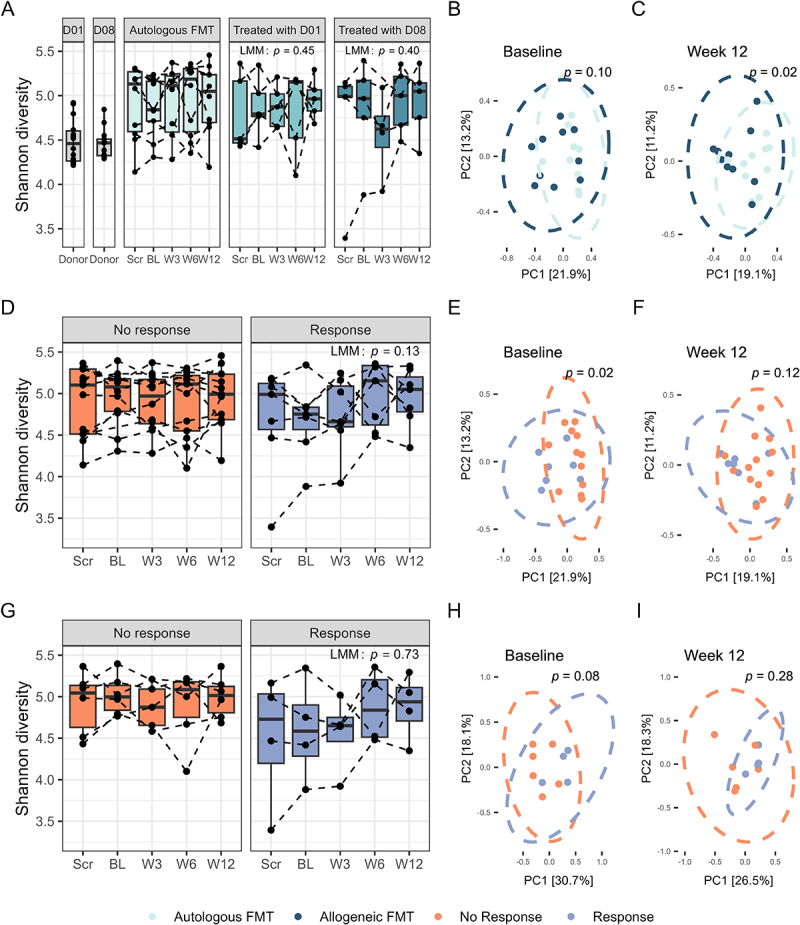
Gut microbiota diversity. A: Shannon diversity (rarefied) over time in patients receiving autologous or allogeneic FMT. B, C: Bray-Curtis dissimilarity in gut microbiota of patients treated with allogeneic versus autologous FMT at baseline (B) and week 12 (C). D, E, F: plots regarding Shannon diversity (rarefied) over time (D), Bray-Curtis dissimilarity at baseline (E), or week 12 (F) separated by clinical response, irrespective of treatment. G, H, I: plots regarding Shannon diversity (rarefied) over time (G), Bray-Curtis dissimilarity at baseline (H), or week 12 (I), separated by clinical response, using only data from patients treated with allogeneic FMT. Panels A, D, and G show *p*-values from linear mixed-effect models (LMM). Panels B, C, E, F, H, and I show *p*-values from PERMANOVA tests. FMT: fecal microbiota transplantation. D01: donor one; D08: donor eight.

Patient microbiota diversity and richness were at or above donor levels at baseline, with no significant differences between treatment or response groups over time (Figure 3A,D,G, Figure S8A-C). However, at week 12, Bray-Curtis-based clustering showed a significant difference in microbiota composition between the treatment groups (*p* = 0.02), primarily driven by D08 (*p* = 0.01), while at baseline, no differences were observed (Figures 3B,C and S9A,B). At baseline, responders and non-responders showed significantly different microbiota composition in Bray-Curtis-based clustering, irrespective of treatment (*p* = 0.02) (Figure 3E). This was not observed post-FMT, nor when the analysis was restricted to responders to allogeneic FMT (Figure 3F,H,I). Microbiota diversity was not significantly associated with continuous changes in HOMA-IR or serum triglycerides (Table S5). Unexpectedly, microbiota diversity showed a stronger negative association with MRI-PDFF in the autologous FMT group, compared to the allogeneic FMT group (Table S5, Figure S10).

Differential abundance analysis revealed no significant differences from baseline in relative abundance at the family, genus, or species levels between treatment or response groups after multiple testing correction (Tables S6–8). However, analysis of relative abundance in relation to individual response parameters showed treatment-dependent associations between two microbial taxa and serum triglyceride levels (Table S9). The genus *Stercorousia* (identified as *Zag1* in GTDB releases ≤R202, belonging to the *Gastranaerophilaceae* family) showed a stronger negative association with triglycerides in the allogeneic FMT group compared to the autologous FMT group, while *Alistipes putredinis* (GTDB R202: *UBA940 sp900768115*, referred to here by its associated National Center for Biotechnology Information name, belonging to the *Rikenellaceae* family) showed a weaker positive association (Figure S11A, B). Both were present in low abundance in donors and patients and were more abundant in D01 compared to D08 (Figure S11C-F).

### Engraftment of the donor microbiota and the course of MASLD-associated bacteria over time

**Figure 4.**
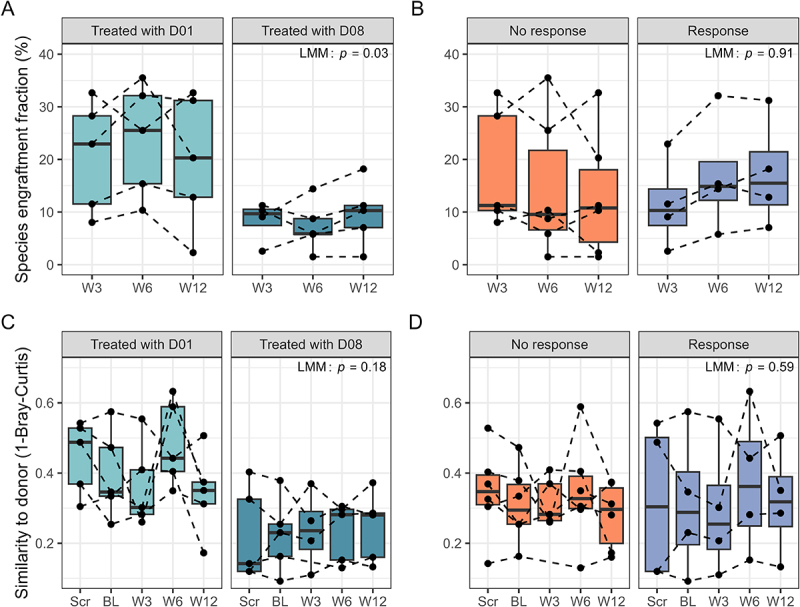
Engraftment of donor gut microbiota. A, B: species level engraftment in patients receiving allogeneic FMT, analyzed by metagenomic sequencing, grouped by FMT donor (A) or response to treatment (B). C, D: mean similarity (1-Bray-Curtis dissimilarity) of patient samples to donor samples over time, grouped by FMT donor (C) and response (D). Differences in species engraftment fraction and mean similarity between treatment and response groups were calculated using linear mixed effect models (LMM). FMT: fecal microbiota transplantation; D01: donor one; D08: donor eight.

Gut microbiota diversity was not significantly different between FMT donors (*p=*0.72), while richness was significantly higher for D01 (*p* < 0.001) (Figure S12A, B). Following FMT, a higher proportion of potentially engraftable donor species were present in patients receiving FMT from D01 compared to D08 (Figure 4A). No significant differences were observed between responders and non-responders, regardless of donor type (Figure 4B). Patient microbiota did not show increased similarity to their respective donors after FMT (Figure 4C,D). Microbiota engraftment and similarity were not significantly associated with individual response parameters (Tables S10, 11). Additionally, no significant differences were observed among bacterial groups and subspecies selected for their potential ethanol-producing capabilities, including *Lactobacillaceae* (*Lactobacillus johnsonii, Ligilactobacillus salivarius, Limosilactobacillus reuteri, Lacticaseibacillus casei*, and *Limosilactobacillus fermentum*), *Streptococcus* (*S. thermophilus*), and *Klebsiella pneumoniae*, which were present in low relative abundance in patients and donors (Figure S13A-G, 14). *A. Muciniphila* relative abundance was lower in patients receiving FMT from D08 compared to those receiving FMT from D01 or autologous FMT at week three (*p=*0.03) and twelve (*p* = 0.01). Feces from D08 showed no detectable *A. Muciniphila* (Figure S13D, H).

## Discussion

To improve the understanding of microbiota modulation for MASLD, we performed a double-blinded RCT, showing no significant effect of consecutive FMT on liver fat content of patients with MASLD. Additionally, we found no significant effects on lipid profiles, liver biochemistry, or glucose tolerance between treatment groups. Clinical response, defined by decreased liver fat, insulin resistance (HOMA-IR) and serum triglycerides, was observed in both the allogeneic (4/10) and autologous (3/10) FMT groups. Patients’ stool microbiota showed high baseline alpha diversity and similar composition across treatment groups, becoming distinct at week 12. No specific microbiota signatures were linked to FMT treatment or response, except for microbial genus *Stercorousia* and *A. putredinis*, which were associated with triglyceride levels following allogeneic FMT. Donor microbiota engraftment appeared donor-specific, but not response-specific.

While animal studies have shown attenuation of liver fat by FMT,^38,39^ the present RCT reports no significant decrease in liver fat 12 weeks after consecutive allogeneic versus autologous FMT in MASLD patients. These results are consistent with two previous small randomized clinical studies comparing allogeneic versus autologous FMT, where one found no significant decrease in MASLD activity score, fibrosis score, and steatosis score in liver biopsies from MASH patients (*n* = 10 versus *n* = 11) at 24 weeks,^20^ and the other found no decrease in MRI-PDFF in MASLD patients (*n* = 15 versus *n* = 6) at 6 months.^25^ Nevertheless, one larger clinical study showed a slight reduction in liver fat by fibroscan 4 weeks after FMT via colonoscopy and enema in 47 patients.^24^ This heterogeneity in clinical results may result from various factors, including differences in donor, patient, and treatment characteristics. In the complex and confounded human model of MASLD, FMT may only have a minimal net effect, suggesting the need for large sample size studies, allowing for patient and donor stratification.

Studies assessing the effect of FMT on liver biochemistry and metabolic parameters show substantial heterogeneity in their outcomes. In accordance with findings in this present study, meta-analyses assessing the effect of FMT on metabolic syndrome and cardiometabolic risk factors have reported no overall difference post-FMT in lipid profiles, glycemic parameters, or anthropometry, despite finding increased HDL and decreased insulin in subgroups.^40,41^ Nevertheless, individual studies report increased insulin sensitivity,^22,23,42^ and decreased HbA1_c_ after FMT.^23,43^ Randomized trials specifically assessing FMT for MASLD found decreases in GGT,^20^ total:HDL cholesterol ratio, and serum non-esterified fatty acids and triglycerides,^25^ but no change in ASAT, ALAT or ALP, total-, HDL-, or LDL-cholesterol, nor insulin sensitivity by HOMA-IR or other measured glycemic parameters.^20,24,25^ Although patients in this present study had elevated PDFF and HOMA-IR at baseline, other parameters were within the normal range, likely explaining their lack of change. Combined with literature, our study indicates that the metabolic effects of FMT in patients with MASLD may be limited and depend on multiple patient, donor and treatment related factors.^25^

Pre-FMT microbiota diversity and richness in MASLD patients were high, contrasting with reports of lower diversity in MASLD^44^ and elevated HOMA-IR.^45^ However, these discrepancies may reflect confounding by microbiota-altering medications in previous studies.^46^ In the present study, no consistent association was observed between microbiota diversity and treatment group or clinical response, despite an unexpected stronger negative association with MRI-PDFF in the autologous versus the allogeneic FMT group. A potential small effect of FMT on microbiota composition was suggested by increased Bray-Curtis dissimilarity between treatment groups at week 12. However, dissimilarity was modest and not related to clinical outcome. Additionally, baseline differences in Bray-Curtis dissimilarity were observed between defined responders and non-responders. While this may theoretically reflect microbiota-factors influencing MASLD outcome,^47^ these findings warrant cautious interpretation given the lack of significant treatment effects and arbitrary, post hoc classification of response.

Associations suggesting potential mitigation of serum triglyceride levels were observed for the *Stercorousia* genus and *A. putredinis*. A recent study reported increased relative abundance of *Stercorousia* in patients not hospitalized for infectious disease compared to those that were, possibly reflecting an association of *Stercorousia* with a less disturbed gut microbiota composition that may be more resistant to infection.^48^ Additionally, biosynthetic gene clusters linked to *Stercorousia* have been negatively associated with BMI.^49^ *A. putredinis* has been negatively associated with MASLD,^50–52^ and was linked to enhanced weight loss from physical activity.^53^ Nevertheless, mechanistic understanding of these taxa and their potential role in lipid metabolism is scarce and warrants further investigation. These exploratory findings should be interpreted with caution as both genera were of low relative abundance. No additional microbial signatures were associated with treatment group or response to treatment, nor were any selected species previously associated with serum ethanol levels in MASLD.^15–18^ The unexpectedly high baseline diversity in patients may have contributed to the overall lack of observed effects, as a diverse microbiota is likely more resistant to disturbance, including to FMT.^54–57^ This aligns with inconsistent findings on microbiota signatures and cardiometabolic risk factors after FMT^41^ and underscores the potential role of baseline recipient factors in FMT efficacy.^47^

Baseline patient and donor factors are likely also important in microbiota engraftment. Pre-FMT patient microbiota diversity and antibiotic treatment pre-FMT may affect engraftment, potentially impacting clinical efficacy across diseases.^57^ In the present study, we found an increased engraftment fraction in patients treated using D01, but no difference between treatment groups or for treatment response. Additionally, the patient microbiota showed no increased resemblance to that of the respective donor after FMT. Interestingly, *A. muciniphila* was detected in D01 but not in D08, possibly reflecting differences in dietary or environmental factors between donors,^58,59^ although no specific anamnestic data were available. Animal studies suggest therapeutic potential for *A. muciniphila* in MASLD and MASH,^60,61^ indicating that its presence in the donor microbiota may provide benefits upon successful engraftment. However, in our cohort, we found no associations with MASLD outcomes, despite a slightly decreased relative abundance at two timepoints after receiving FMT from D08. Although the overall lack of association between engraftment and clinical outcome may be due to the highly diverse pre-FMT patient microbiota, the role of donor selection and microbiota engraftment in MASLD remains unexplored and warrants further investigation.

Limitations of this study include its limited sample size for detecting small effects and for exploratory analyses of secondary outcomes, microbiota profiles, and interaction effects in LMMs. Additionally, future studies could reduce pre-FMT patient microbiota diversity by patient selection or antibiotic pre-treatment.^56^ As studies found heterogeneous outcomes in measures of liver fat content and insulin sensitivity at follow-up both shorter and longer than 12 weeks,^20,22–25^ measuring MRI-PDFF and metabolic parameters at additional time points could capture transient or delayed effects. Finally, although this study provided dietary and lifestyle advice to patients, dietary intake and lifestyle were not monitored, complicating confounder correction. Active lifestyle and improved diet quality, such as a Mediterranean-style diet, can prevent and ameliorate MASLD, mediated through improvements in insulin sensitivity and lipid metabolism, potentially in part through modulation of the gut microbiota.^8,62–64^

In conclusion, this longitudinal, double-blinded randomized controlled trial found no significant effect of allogeneic versus autologous FMT on liver fat content, liver biochemistry, glycemic parameters, or lipid profiles in patients with MASLD. Combined clinical response to treatment was observed equally in both treatment groups. At week 12, patients treated with allogeneic FMT had a different microbiota composition compared to those receiving autologous FMT. Although exploratory analyses identified associations between serum triglyceride levels and two specific bacterial taxa, neither FMT nor clinical response was linked to a specific microbiota signature or donor microbiota engraftment. Future studies should stratify patients by microbiota diversity, explore recipient-specific factors influencing FMT efficacy, or consider pre-FMT antibiotic treatment.

## Supplementary Material

250406_Supplement 1 supplementary methods.docx

250630_Supplement 2 supplementary figures_revised.docx

250406_Supplement 3 supplementary tables.docx

## Data Availability

The raw metagenomic data for this report are available in the National Center for Biotechnology Information Sequence Read Archive under BioProject number: PRJNA1251999 (https://www.ncbi.nlm.nih.gov/bioproject/PRJNA1251999).
