# Supplementary material for: Consecutive fecal microbiota transplantation for metabolic dysfunction-associated steatotic liver disease: a randomized controlled trial
Source: Gut Microbes. 2025 Aug 4;17(1):2541035. doi: 10.1080/19490976.2025.2541035 (PMC12323438; doi:10.1080/19490976.2025.2541035)
Supplement: 250406_Supplement 1 supplementary methods.docx [file KGMI_A_2541035_SM3900.docx]

**Supplementary methods**

Exclusion criteria

| - Exclusion criteria for MRI (claustrophobia, pacemaker, metal implants, etc). |
| --- |
| - Any other liver disease than MASLD/MASH. |
| - Present excessive alcohol use defined as > 2 units/day. |
| - Recent use (< 3 months) of antibiotics. |
| - Use of possible drugs interfering microbiota or recent (< 3 months) changes in dosages |
| - Recent (< 3 months) weight change (>5%) |
| - Cardiovascular co-morbidity defined as heart failure, coronary insufficiency and hypertension in past history. |
| - Previous use of glucocorticosteroids, hormonal substitution, pagitaxel, theofyllin, amiodarone, myelosuppresive agents. |
| - A psychiatric, addictive or any other disorder that compromises the subjects ability to understand the study content and to give written informed consent for participation in the study. |

Sample size calculation

The formal sample size calculation was based on prior studies examining liver fat content (1, 2). Detecting a 5% difference in liver fat content with a power of 0.8 and confidence level of 0.95 was calculated to require at least 9 subjects per group. Consequently, 10 subjects per treatment group were included in this study.

Missing data

Missing measurements in OGTT time-series (0-120 minutes) were imputed 100 times with 20 iterations using the MICE package (v3.17.0). When >2 consecutive measurements were missing in a time-series, data was not imputed. Areas under the curve (AUCs) and incremental AUCs (iAUCs) were calculated per visit. Statistical results from linear mixed effects models s were pooled using Rubin’s formulae.

Metagenomic analyses

*Species engraftment fraction (SEF)*

SEF was calculated as described in van Lingen et al. (3), with minor adaptations. Core donor species were defined as those detected in ≥10 samples out of 14 or 13 for donor D01 and D08, respectively. Donors D01 and D08 had core microbiota of 416 and 330 species, respectively. Total relative abundance of core donor species always accounted for >90% of the total relative species abundance in each individual sample. SEF was calculated by: 1) defining a list of “engraftable donor species” by excluding species overlapping between the core donor microbiota and the pre-FMT patient microbiota, 2) determining the number of engraftable donor species detected in the recipient post-FMT, and 3) dividing this by the number of engraftable donor species (3). The median number of potentially engrafted species per recipient was 98 [range: 68–156].

*Microbiota similarity*

Similarity between patient and respective donor microbiota was calculated by comparing species compositions, using an adaptation to the method described by van Lingen et al (3). Mean Bray-Curtis dissimilarity to the corresponding donor microbiota was calculated. Values were converted to similarity by performing: 1-dissimilarity.

Linear mixed effects models used

*Parameters used:*

- alias = patient alias
- **type_of_fmt = 0, 1**
- **treatment** = 1 if type_of_fmt = 1 and timepoint ≠ baseline
- time = 0 at baseline and 1 after intervention
- responder = 0, 1
- response = 1 if responder = 1 and timepoint ≠ baseline
- response_parameter = either HOMA-IR, MRI-PDFF, or serum triglycerides

*Models for analysis of differences in secondary outcomes between treatment groups (lme4):*

- non-baseline_value_of_interest ~ baseline_value_of_interest + type_of_fmt + (1|alias)

*Additional models for microbiota parameters (lme4):*

- Analysis of longitudinal associations between microbiota parameters and response, irrespective of treatment.
  - non-baseline_value_of_interest ~ baseline_value_of_interest + responder + (1|alias)
- Analysis of longitudinal associations between microbiota parameters and response parameters, irrespective of treatment.
  - delta_value_of_interest ~ baseline_value_of_interest + baseline_response_parameter + delta_response_parameter + type_of_fmt + (1|alias)
- Analysis of longitudinal associations between microbiota parameters and response parameters, and how these associations differ between treatment groups.
  - delta_value_of_interest ~ baseline_value_of_interest + baseline_response_parameter + delta_response_parameter*type_of_fmt + (1|alias)

*Models for differential abundance analysis (LinDA) (4):*

- Analysis of longitudinal differences in relative abundance between treatment groups
  - relative_abundance ~ treatment + time + (1+time|alias)
- Analysis of longitudinal differences in relative abundance between response groups, irrespective of treatment
  - relative_abundance ~ response + time + (1+time|alias)
- Analysis of longitudinal associations between relative abundance and response, and how these associations differ between treatment groups
  - relative_abundance ~ time + treatment*response + (1+time|alias)
- Analysis of longitudinal associations between relative abundance and individual response parameters, and how these associations differ between treatment groups
  - relative_abundance ~ time + response_parameter*treatment + (1+time|alias)

**References**

1. Seppälä-Lindroos A, Vehkavaara S, Häkkinen AM, Goto T, Westerbacka J, Sovijärvi A, et al. Fat accumulation in the liver is associated with defects in insulin suppression of glucose production and serum free fatty acids independent of obesity in normal men. J Clin Endocrinol Metab. 2002;87(7):3023-8.

2. Tiikkainen M, Bergholm R, Vehkavaara S, Rissanen A, Häkkinen AM, Tamminen M, et al. Effects of identical weight loss on body composition and features of insulin resistance in obese women with high and low liver fat content. Diabetes. 2003;52(3):701-7.

3. van Lingen E, Nooij S, Terveer EM, Crossette E, Prince AL, Bhattarai SK, et al. Faecal Microbiota Transplantation Engraftment After Budesonide or Placebo in Patients With Active Ulcerative Colitis Using Pre-selected Donors: A Randomized Pilot Study. Journal of Crohn's and Colitis. 2024;18(9):1381-93.

4. Zhou H, He K, Chen J, Zhang X. LinDA: linear models for differential abundance analysis of microbiome compositional data. Genome Biology. 2022;23(1):95.
