# Supplementary material for: Consecutive fecal microbiota transplantation for metabolic dysfunction-associated steatotic liver disease: a randomized controlled trial
Source: Gut Microbes. 2025 Aug 4;17(1):2541035. doi: 10.1080/19490976.2025.2541035 (PMC12323438; doi:10.1080/19490976.2025.2541035)
Supplement: 250630_Supplement 2 supplementary figures_revised.docx [file KGMI_A_2541035_SM3899.docx]

**
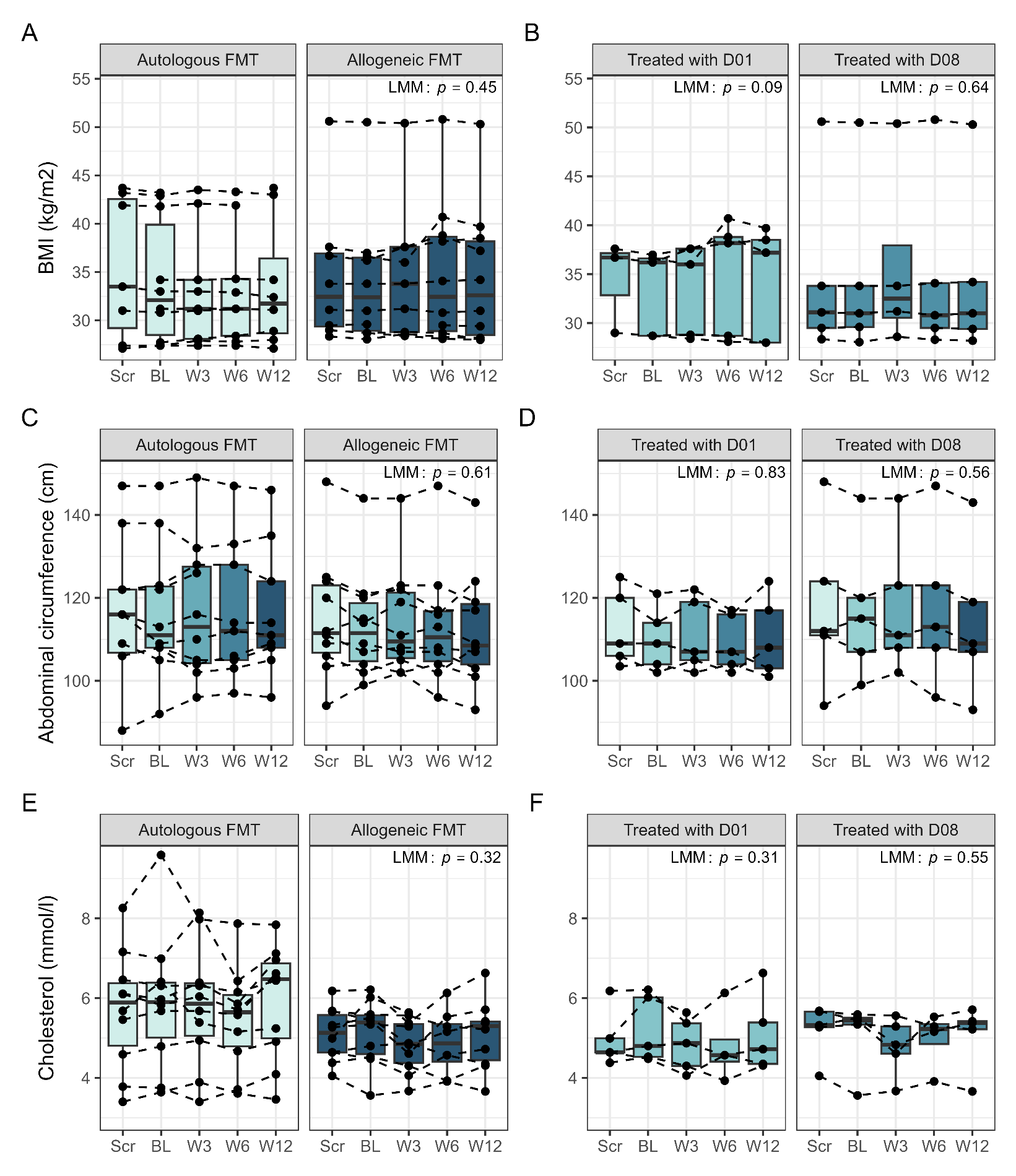
Supplementary figure 1. Body mass index (BMI), abdominal circumference and cholesterol over time, separated by intervention group.** FMT: faecal microbiota transplantation; D01: donor one; D08: donor eight; LMM: linear mixed effects model.

**
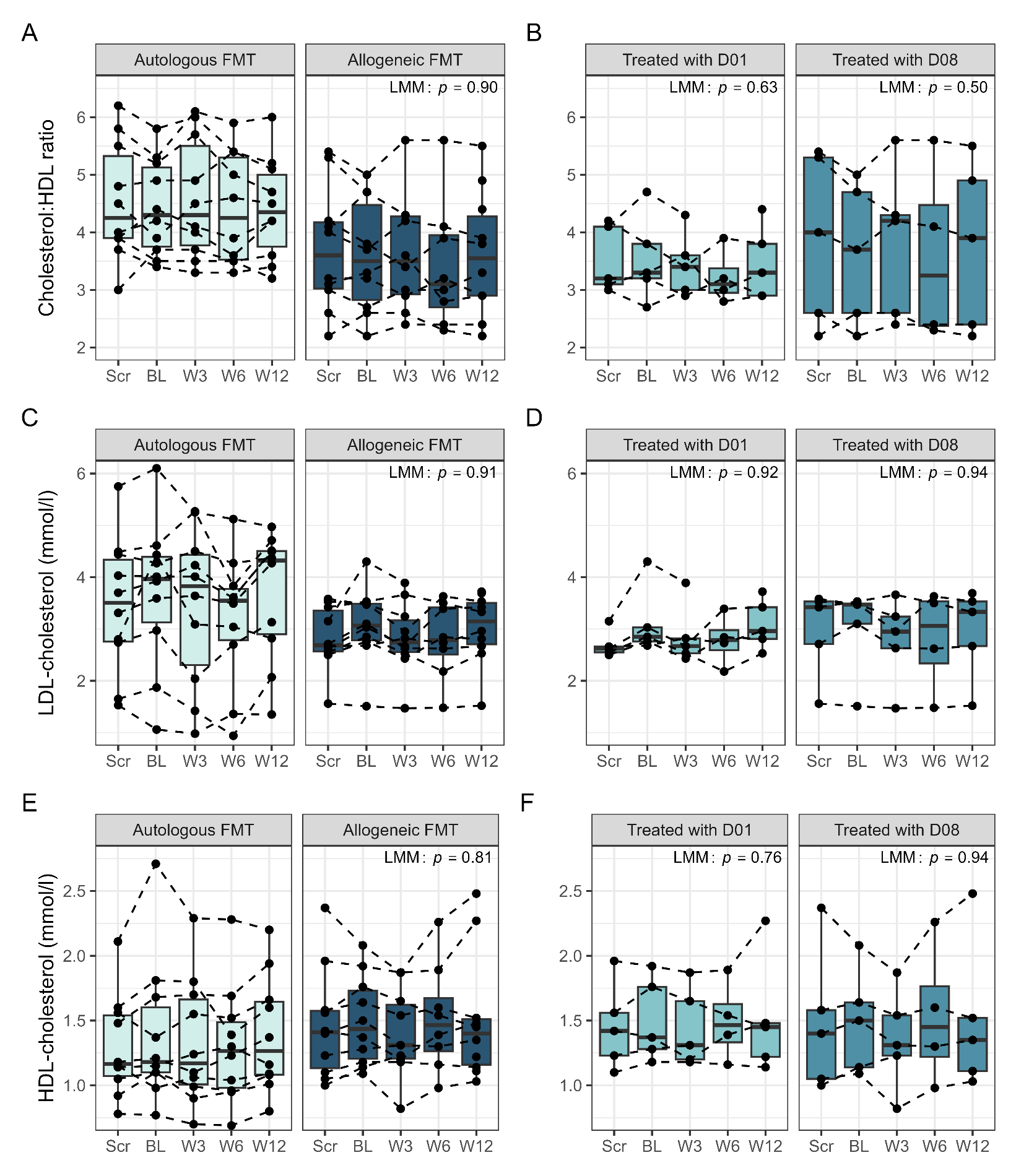
Supplementary figure 2. LDL and HDL cholesterol over time, separated by intervention group.** FMT: faecal microbiota transplantation; D01: donor one; D08: donor eight; LMM: linear mixed effects model; HDL: high-density lipoprotein; LDL: low-density lipoprotein.

**Supplementary figure 3.
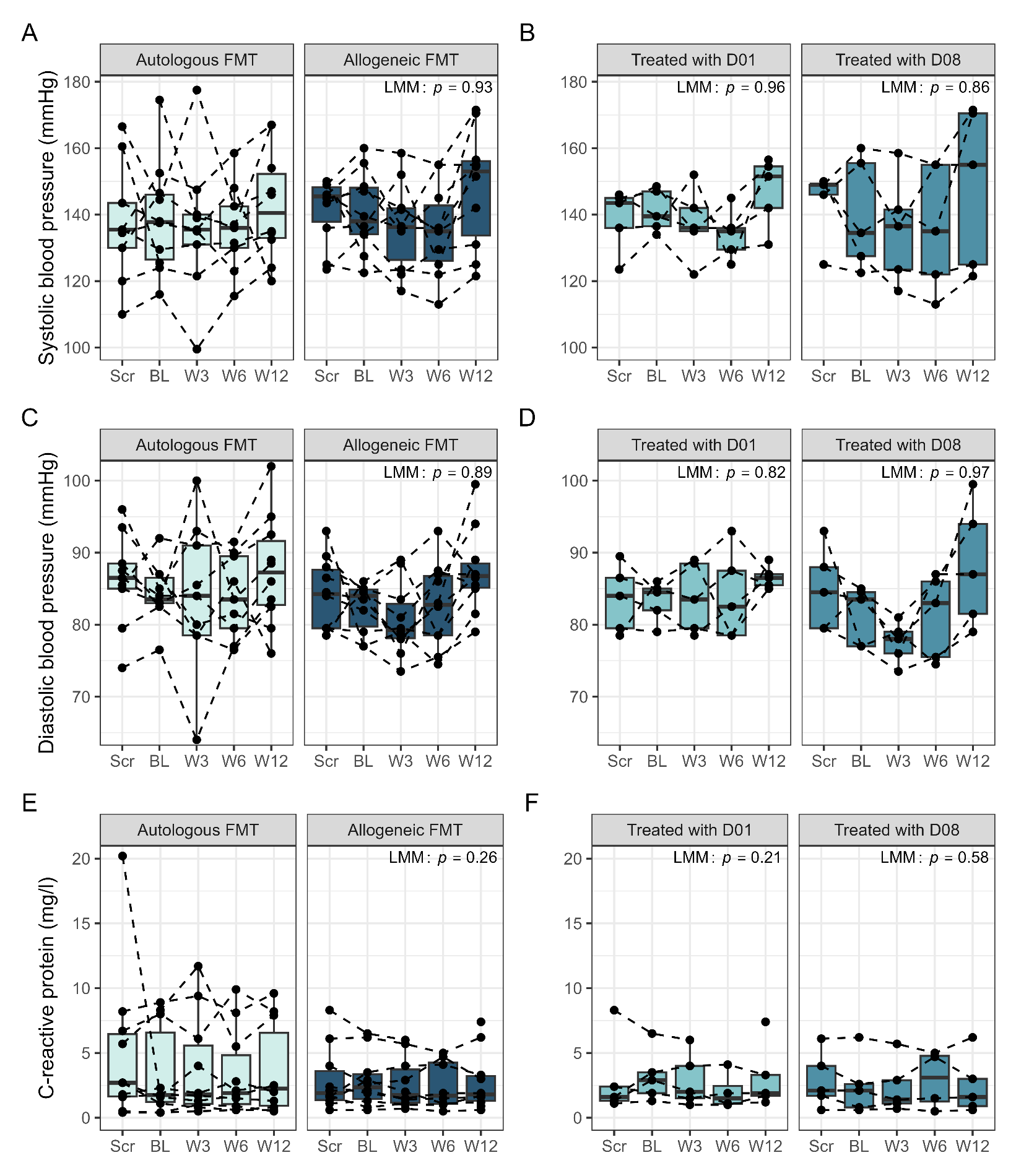
Blood pressure and c-reactive protein over time, separated by intervention group.** FMT: faecal microbiota transplantation; D01: donor one; D08: donor eight; LMM: linear mixed effects model.

**
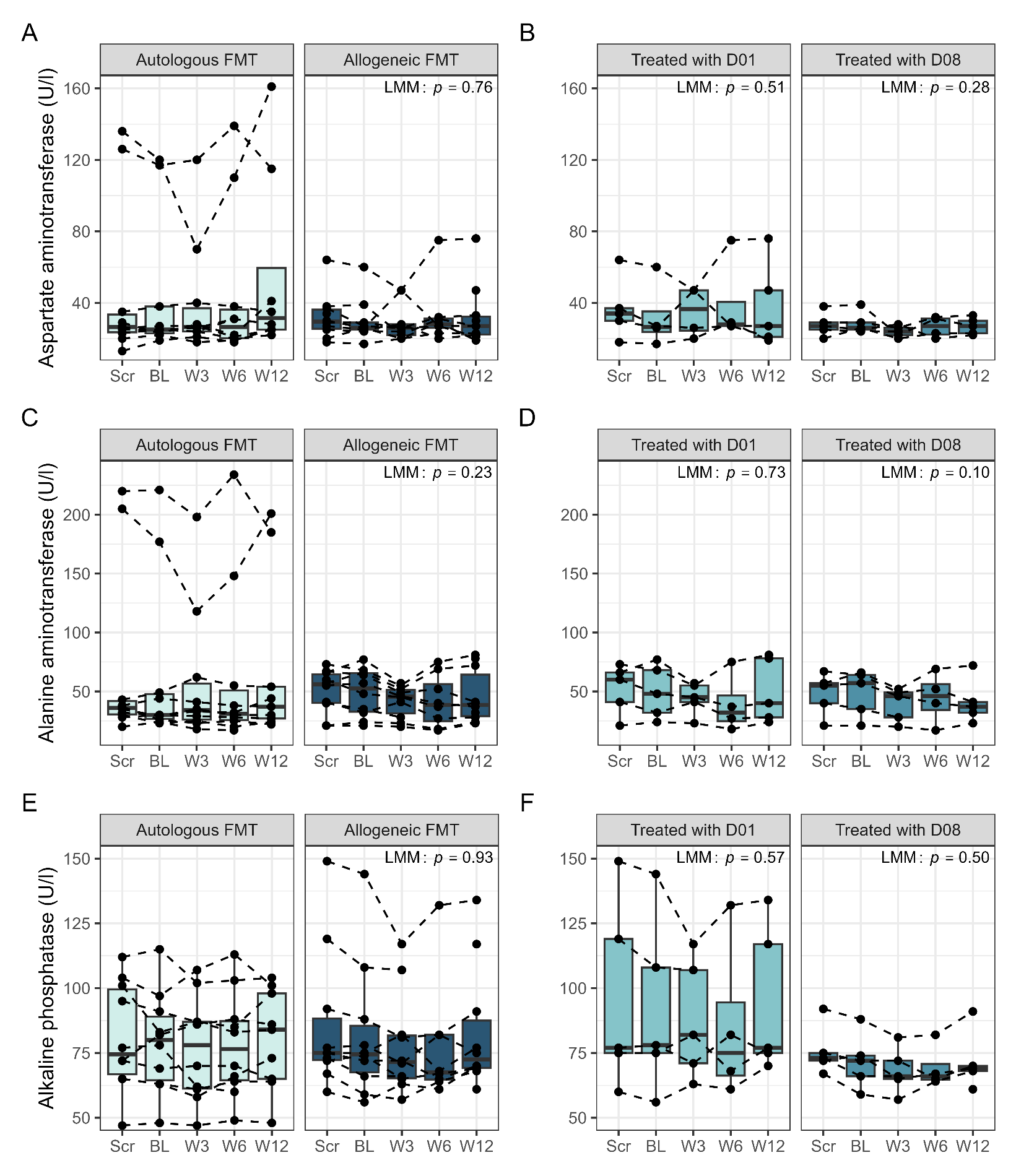
Supplementary figure 4. Liver biochemistry over time, separated by intervention group.** FMT: faecal microbiota transplantation; D01: donor one; D08: donor eight; LMM: linear mixed effects model.

**
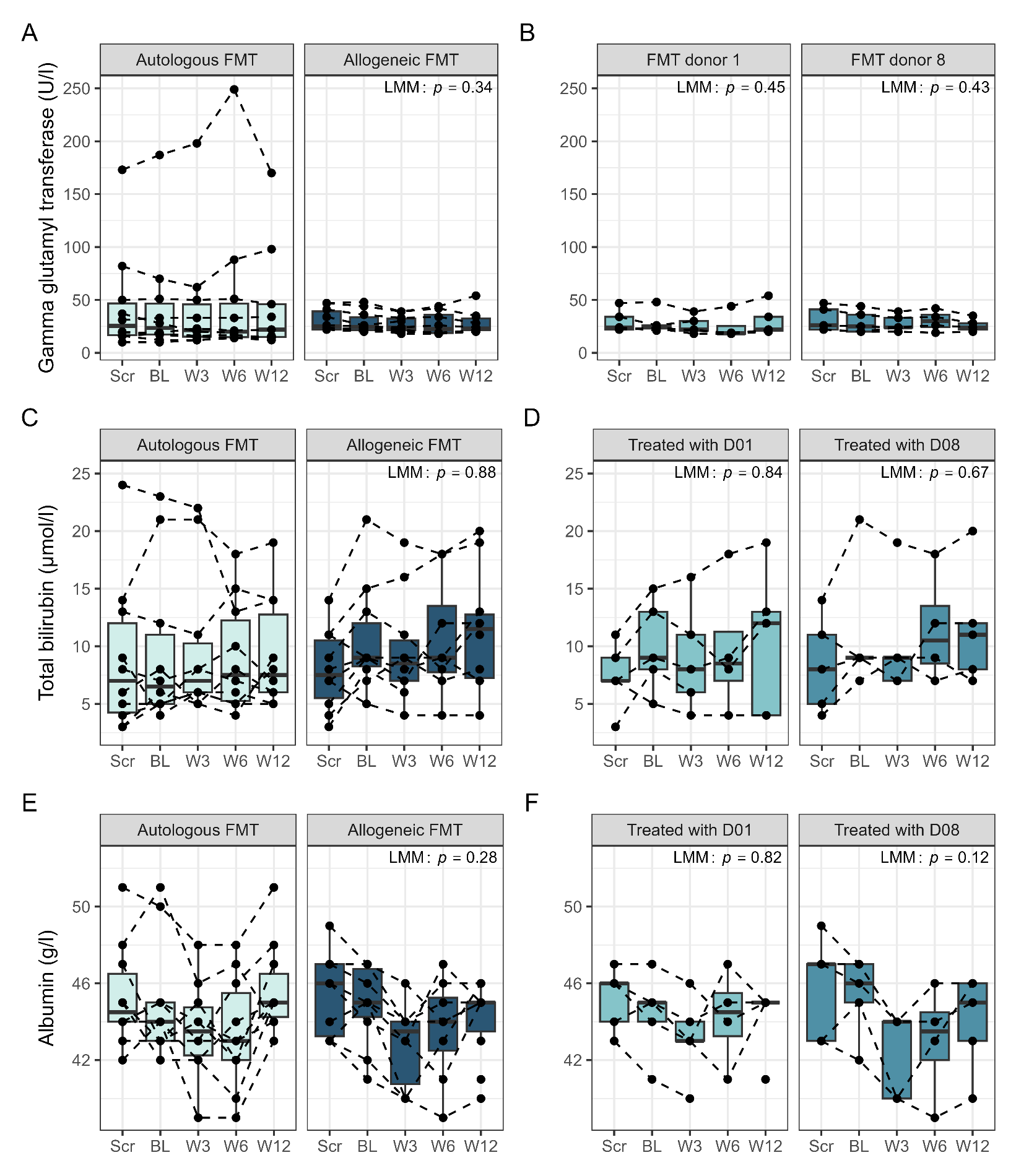
Supplementary figure 5. Liver biochemistry and albumin over time, separated by intervention group.** FMT: faecal microbiota transplantation; D01: donor one; D08: donor eight; LMM: linear mixed effects model.

**
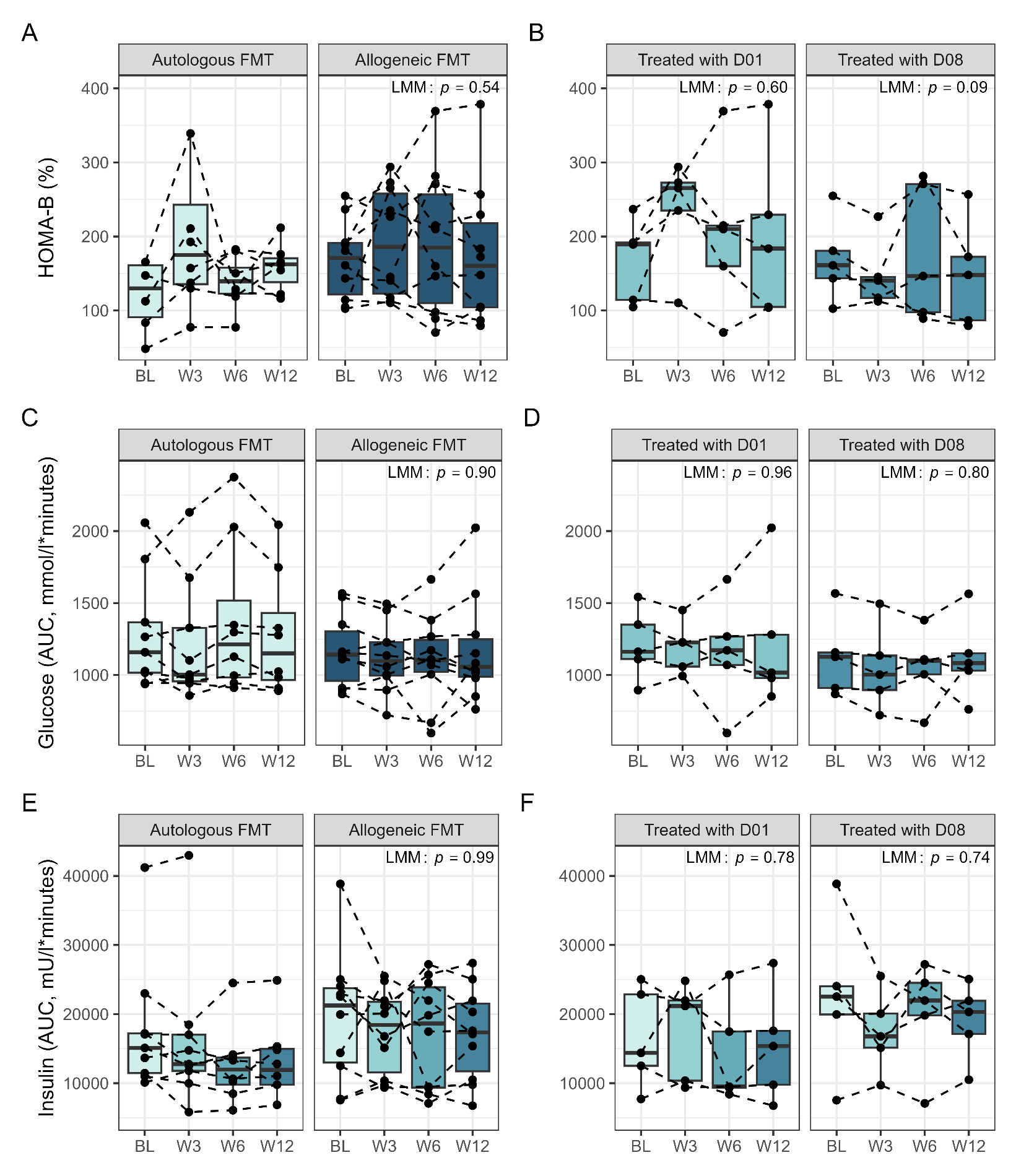
Supplementary figure 6. HOMA-B and glucose and insulin AUCs over time, separated by intervention group.** FMT: faecal microbiota transplantation; D01: donor one; D08: donor eight; LMM: linear mixed effects model; HOMA-B: homeostatic model assessment of β-cell function, AUC: area under the curve obtained from seven-point oral glucose tolerance tests (0-120 minutes).

**
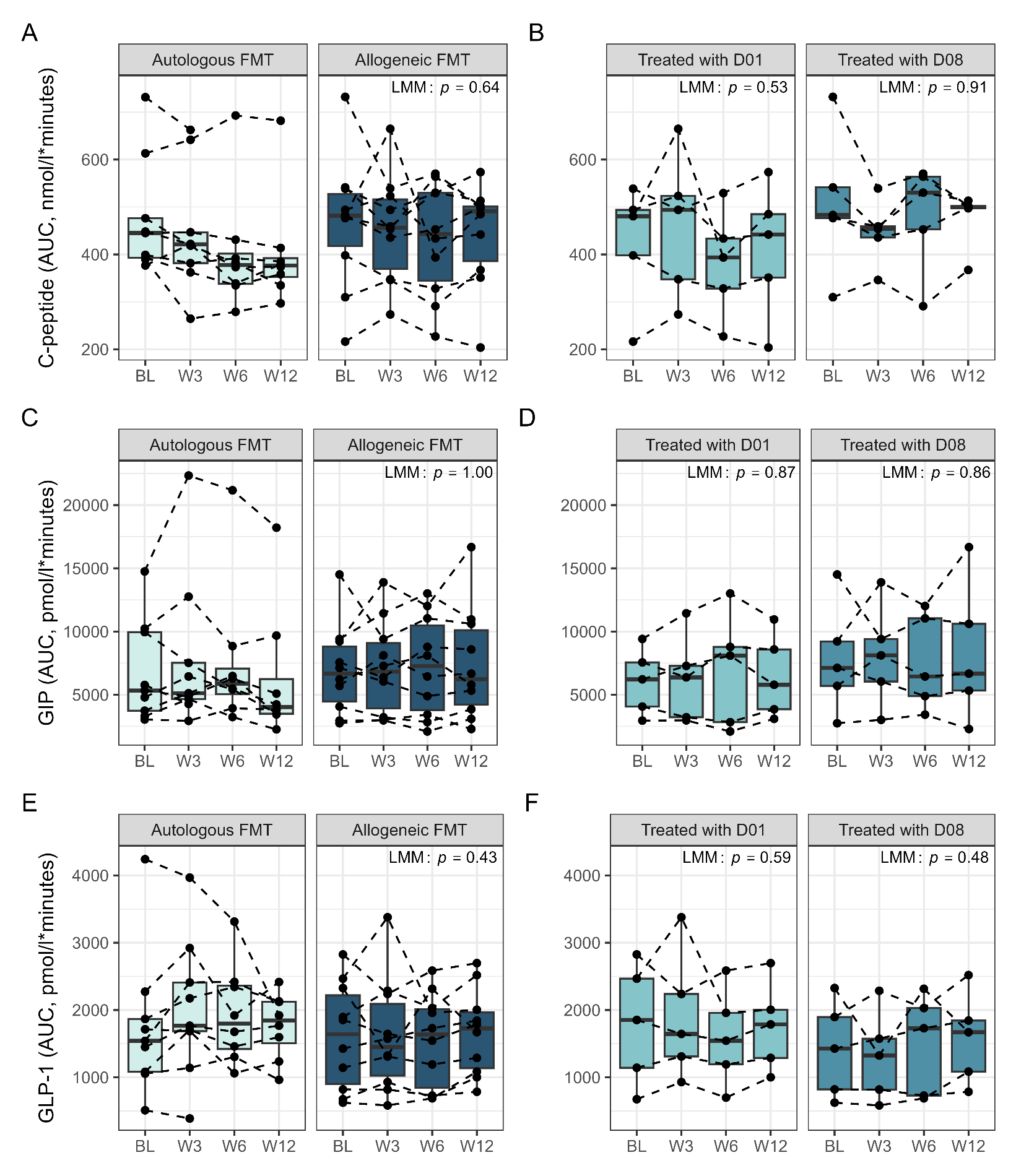
Supplementary figure 7. C-peptide and incretin AUCs over time, separated by intervention group.** FMT: faecal microbiota transplantation; D01: donor one; D08: donor eight; LMM: linear mixed effects model; GIP: gastric-inhibitory polypeptide; GLP-1: glucagon-like peptide-1 AUC: area under the curve obtained from seven-point oral glucose tolerance tests (0-120 minutes).

**
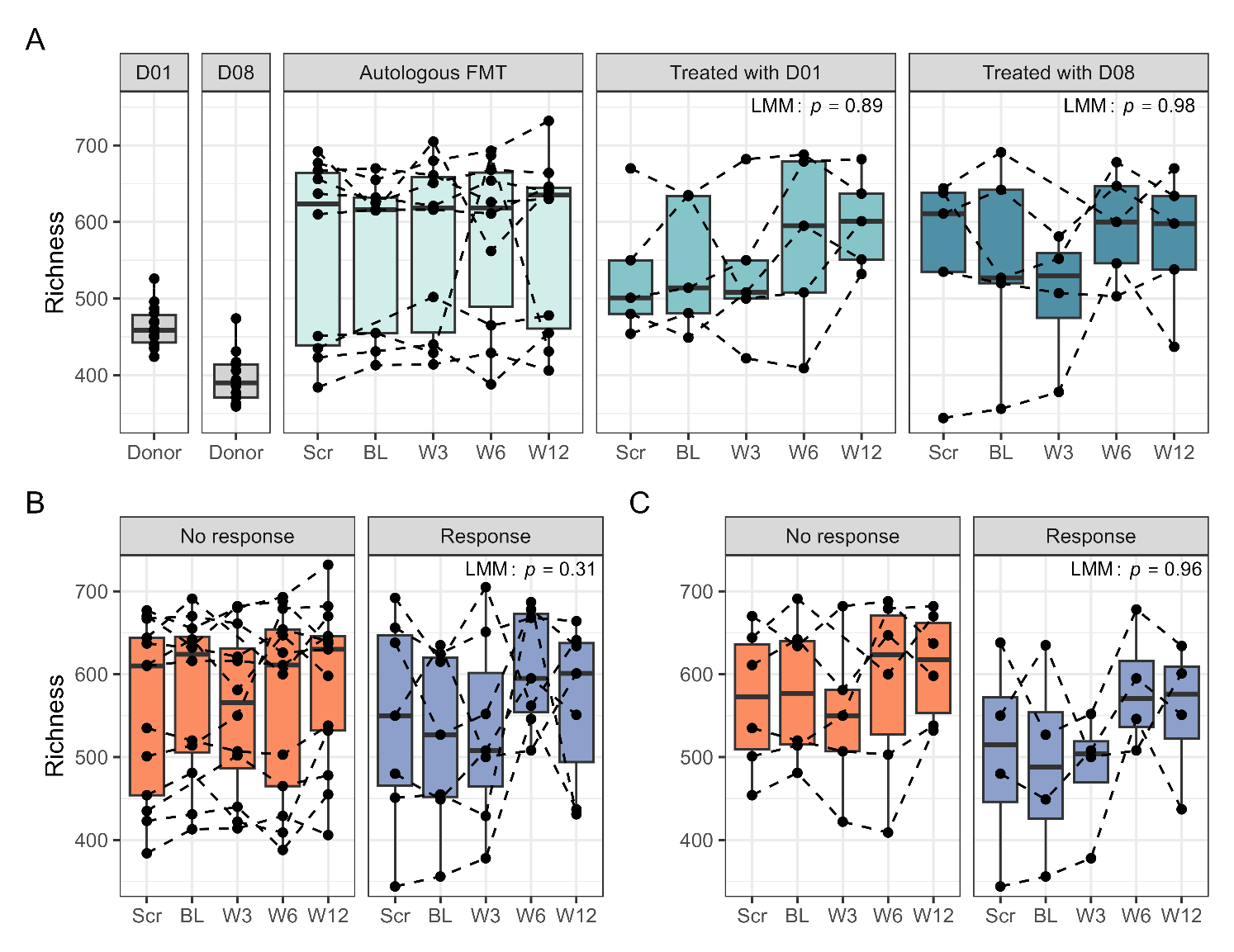
Supplementary figure 8. Gut microbiota richness over time, separated by intervention group and clinical response.** Clinical response was defined as any decrease in magnetic resonance imaging-derived proton density fat fraction (MRI-PDFF), homeostatic model assessment for insulin resistance (HOMA-IR), and serum triglycerides at week 12. A: microbial richness split per FMT donor. B, C: microbial richness for defined responders and non-responders. FMT: faecal microbiota transplantation; D01: donor one; D08: donor eight; LMM: linear mixed effects model.

**
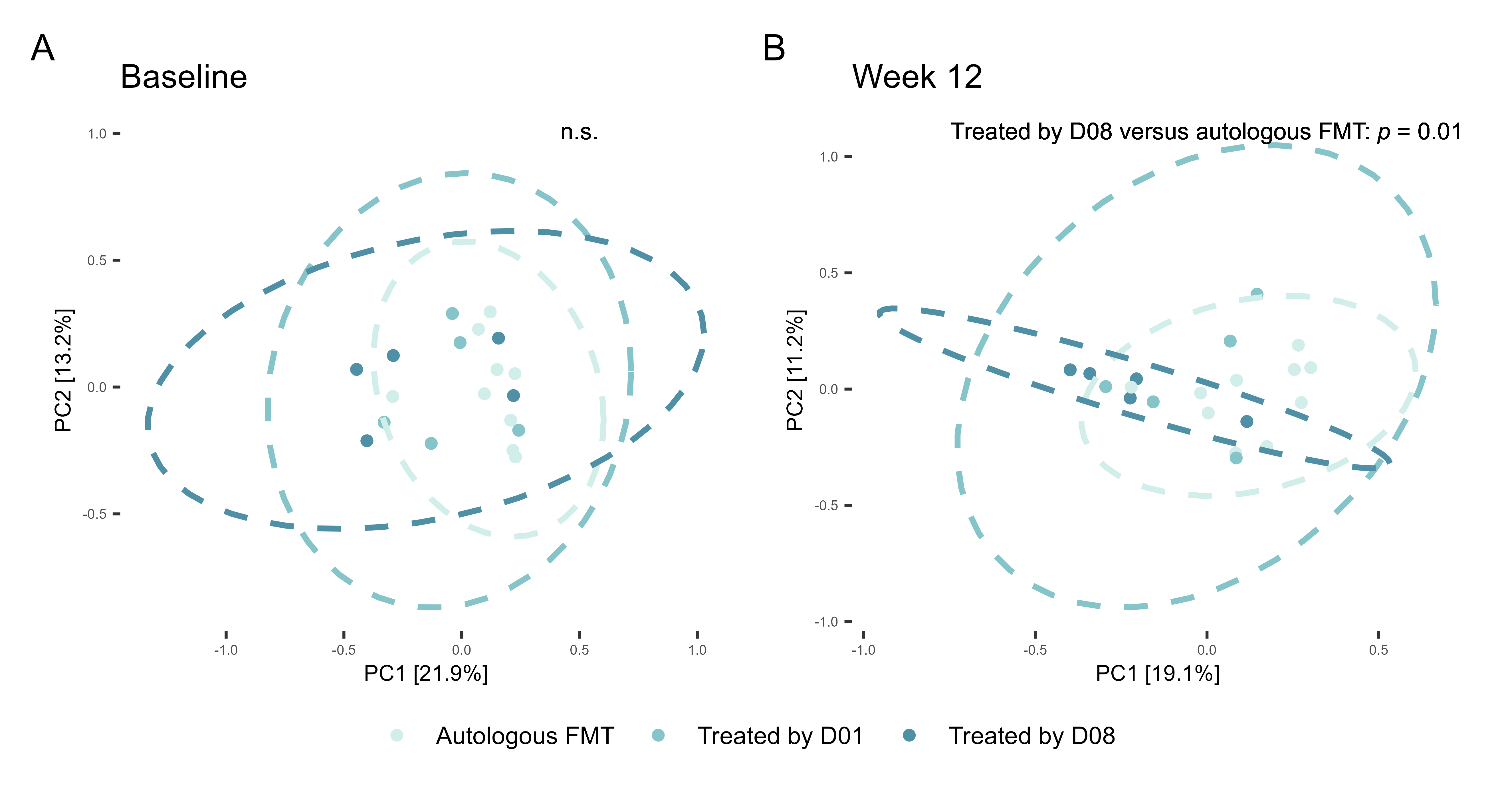
Supplementary figure 9. Beta diversity separated by FMT donor.** Beta diversity (Bray-Curtis) separated by the specific donor used for treatment at baseline (A) and week 12 (B). FMT: faecal microbiota transplantation; D01: donor one; D08: donor eight; LMM: linear mixed effects model.

**
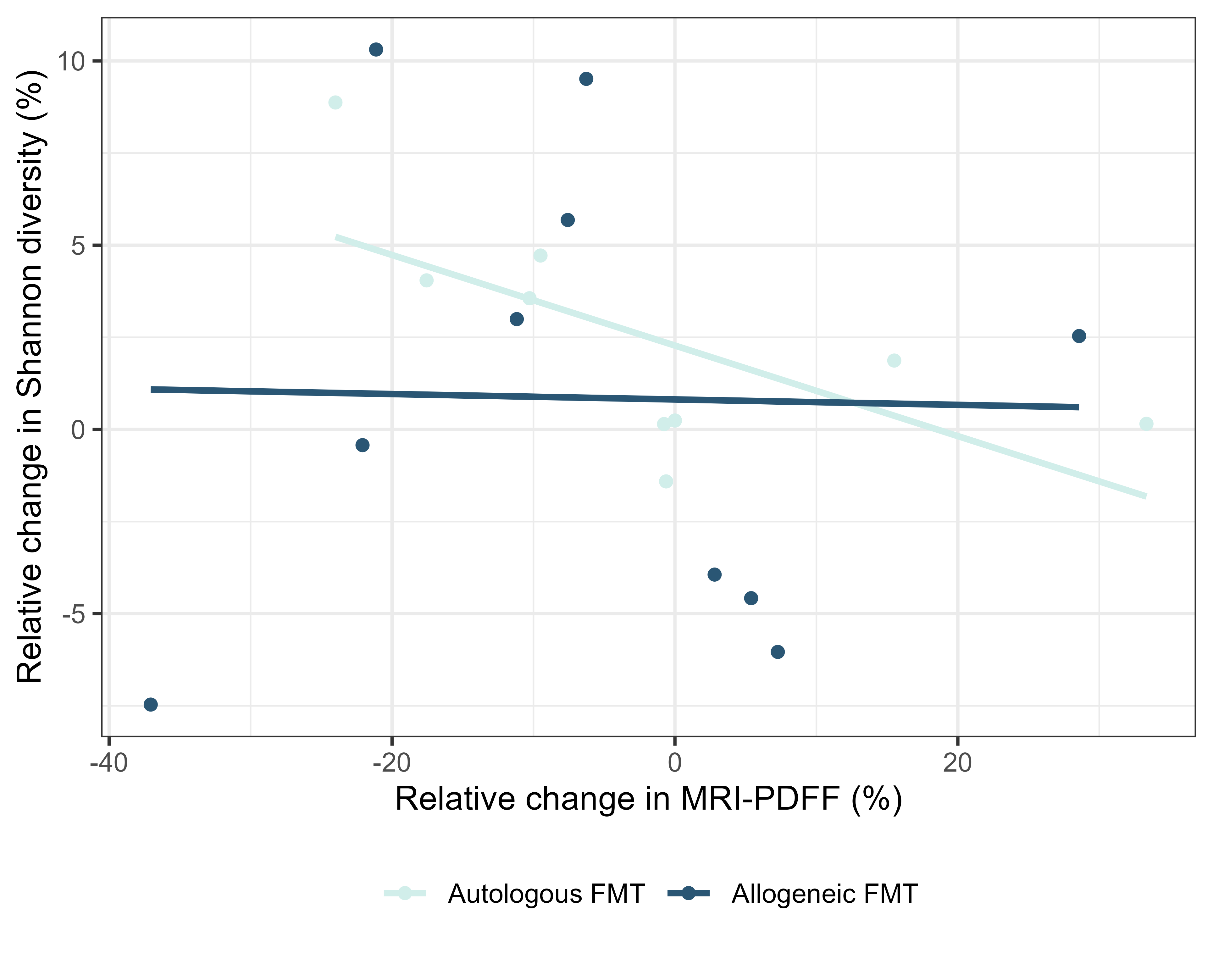
Supplementary figure 10. Association between relative changes in Shannon diversity and MRI-PDFF across FMT treatment groups.** Relative change in Shannon diversity was calculated as the percentage difference between baseline and the mean of measurements at post-FMT timepoints (week 3, 6, 12)**.** Relative change in MRI-PDFF was calculated as the percentage difference between timepoints baseline and week 12. Data was fitted with linear functions per treatment group for visual purposes. FMT: faecal microbiota transplantation; MRI-PDFF: magnetic resonance imaging-derived proton density fat fraction.


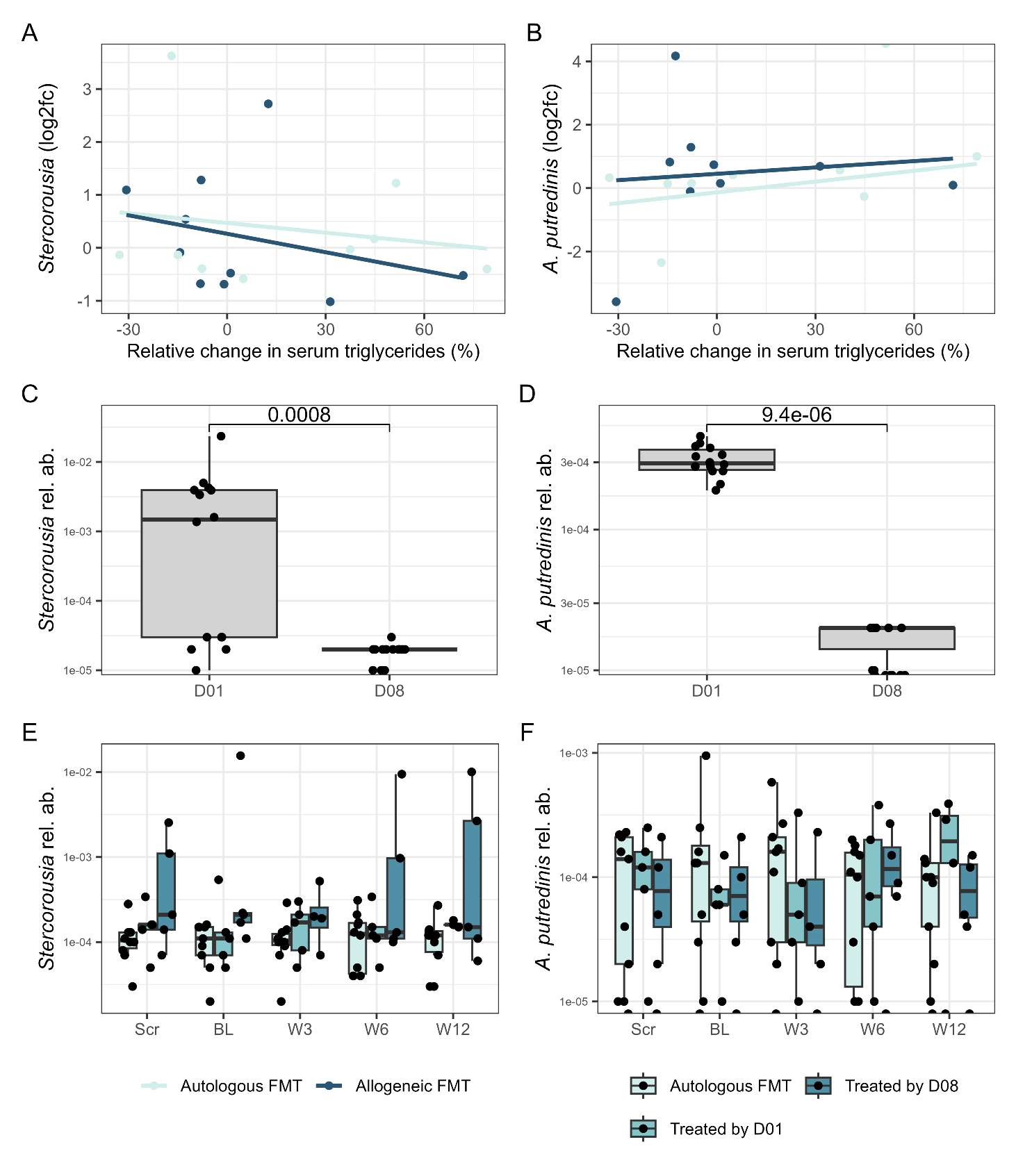
**Supplementary figure 11. Presence of the genus** ***Stercorousia* and *Alistipes putredinis* in donors and patients and associations with serum triglyceride levels.** A, B: The log2 fold change in relative abundance of Stercorousia and *A. putredinis* from baseline to post-FMT (mean relative abundance of weeks 3, 6 and 12) was plotted against the relative change in serum triglycerides over the same period, stratified by FMT treatment group to evaluate associations between relative abundance and serum triglyceride levels, and assess their variation across treatment groups. C, D: Relative abundance of Stercorousia and *A. putredinis* per donor. *P-*values represent Wilcoxon rank-sum tests. E, F: Relative abundance of Stercorousia and *A. putredinis* in patients over time. FMT: faecal microbiota transplantation; D01: donor one; D08: donor eight; log2fc: log2 fold change; rel. ab.: relative abundance. *Stercorousia* and *A. putredinis* (associated NCBI nomenclature) were respectively defined *Zag1* and *UBA940 sp900768115* in GTDB R202.


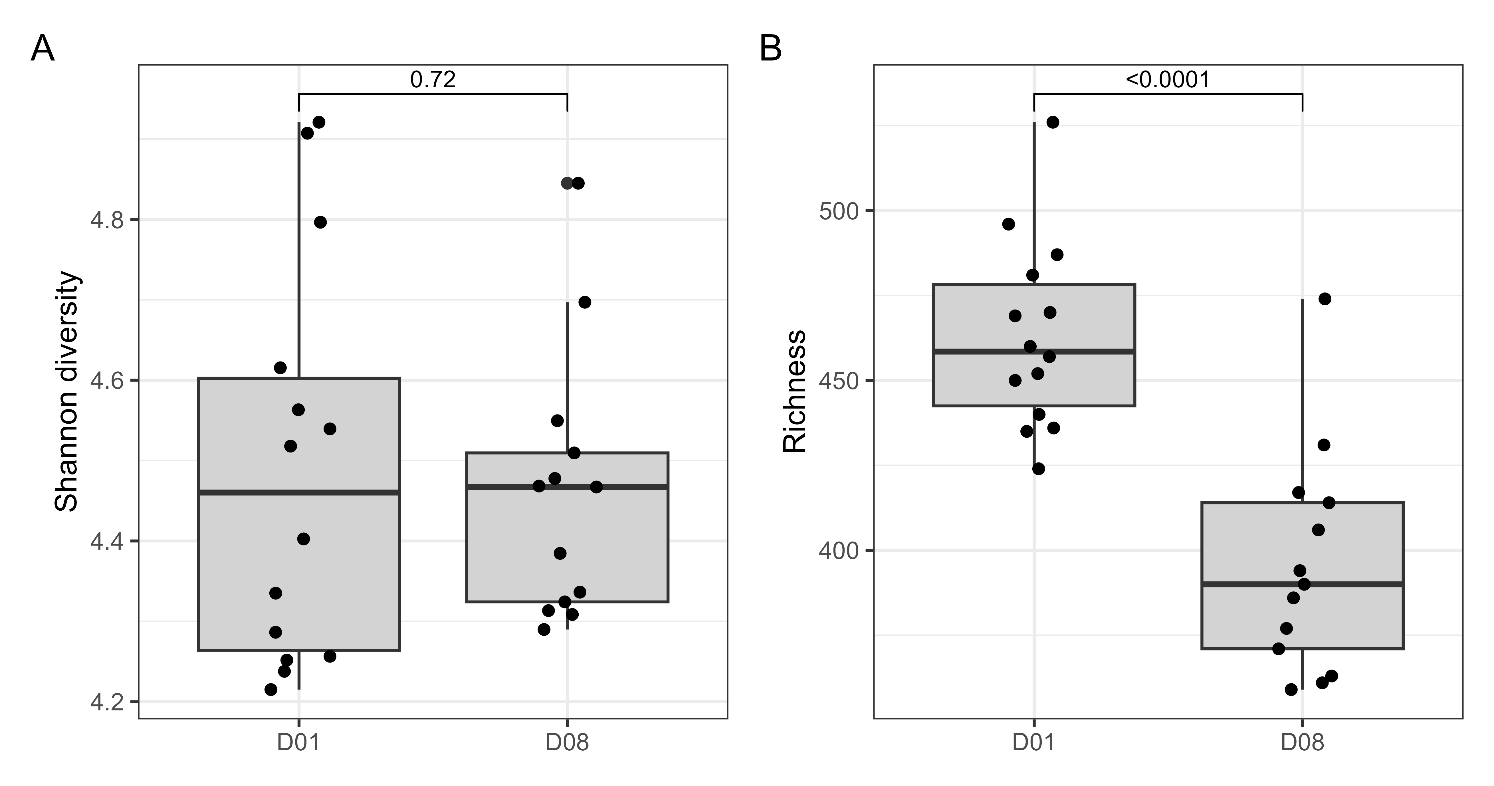
**Supplementary figure 12. Microbiota diversity and richness of donor microbiota in samples used for faecal microbiota transplantation.** A: Diversity (Shannon, rarefied). B: Richness (rarefied). D01: donor one; D08: donor eight.


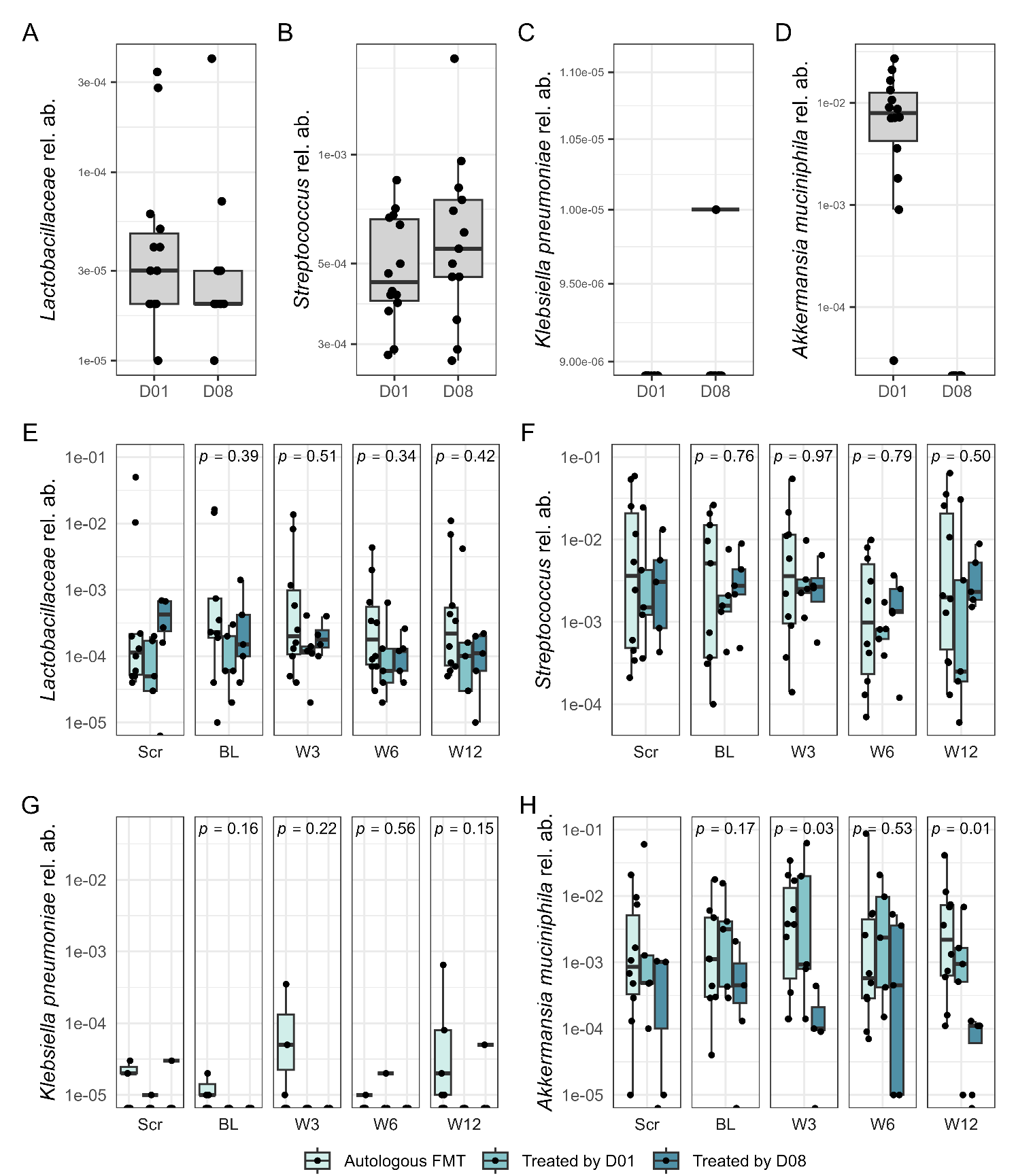


**Supplementary figure 13 Relative abundance of selected bacterial families and genera over time.** A-D: comparison of relative abundances of *Lactobacillaceae, Streptococcus, Klebsiella pneumoniae,* and *Akkermansia muciniphila* present in FMT donor samples. E-H: Relative abundances of *Lactobacillaceae, Streptococcus, K. pneumoniae,* and *A. muciniphila* per treatment group over time, *p-*values represent Kruskal-Wallis tests per timepoint. FMT: faecal microbiota transplantation; D01: donor one; D08: donor eight; rel. ab.: relative abundance.


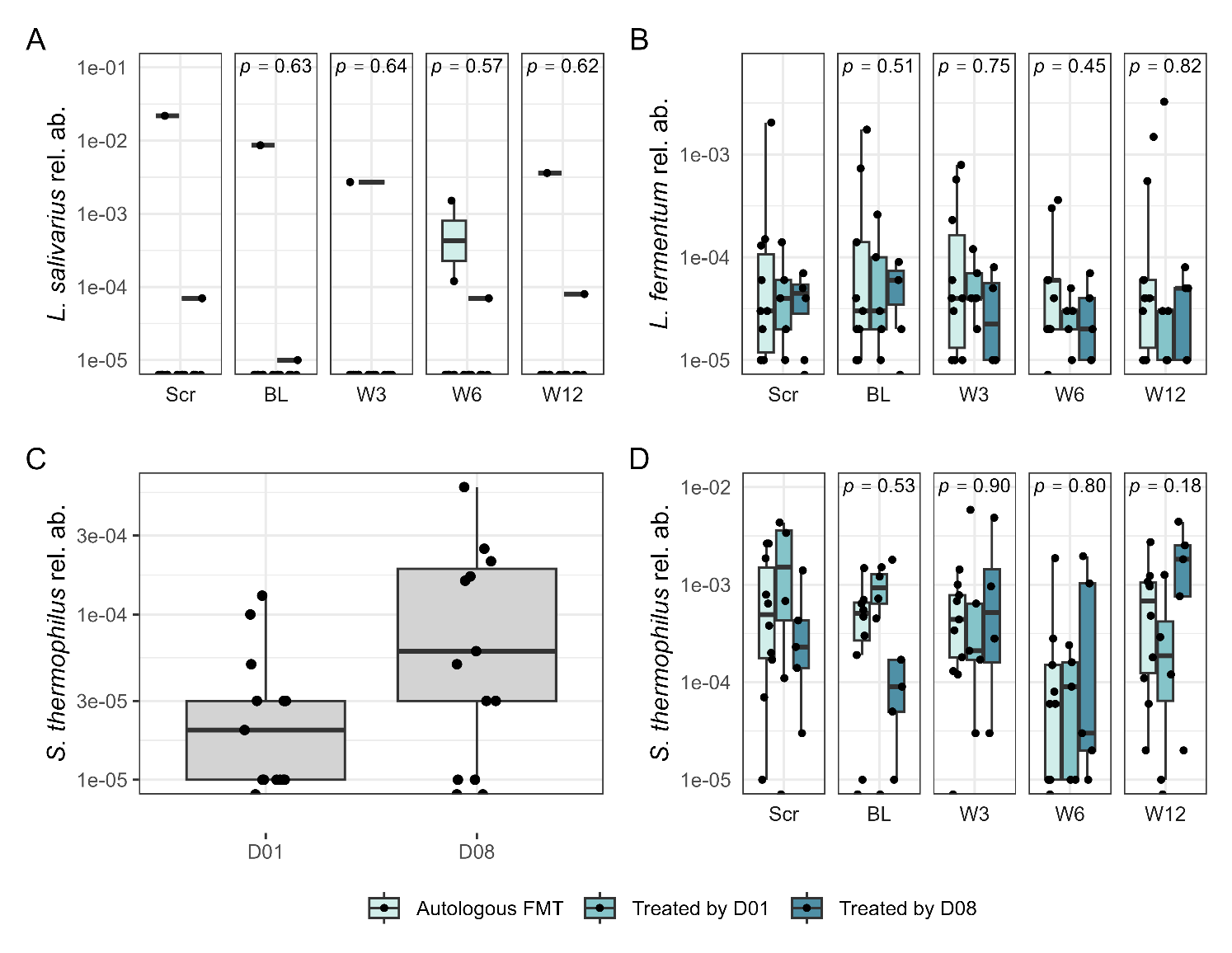
**Supplementary figure 14 Relative abundance of selected *Lactobacillaceae* subspecies and *Streptococcus thermophilus*.** A, B: Out of Lactobacillaceae species of interest for their potential ethanol producing capabilities (Lactobacillus johnsonii, Ligilactobacillus salivarius, Limosilactobacillus reuteri, Lacticaseibacillus casei, and Limosilactobacillus fermentum) only *L. salivarius* (A) and *L. fermentum* (B) were reliably detected in patient faeces and depicted over time. None of these *Lactobacillaceae* species were reliably detected in donor faeces. C: Relative abundance of *S. thermophilus* in donor samples. D: Relative abundance of *S. thermophilus* in patient samples over time. All *p-*values represent Kruskal-Wallis tests per timepoint. FMT: faecal microbiota transplantation; D01: donor one; D08: donor eight; rel. ab.: relative abundance.
