# Supplementary material for: Consecutive fecal microbiota transplantation for metabolic dysfunction-associated steatotic liver disease: a randomized controlled trial
Source: Gut Microbes. 2025 Aug 4;17(1):2541035. doi: 10.1080/19490976.2025.2541035 (PMC12323438; doi:10.1080/19490976.2025.2541035)
Supplement: 250406_Supplement 3 supplementary tables.docx [file KGMI_A_2541035_SM3898.docx]

**Supplementary table 1. Effects of consecutive allogeneic FMT on anthropometric parameters, liver enzymes and bilirubin over time.**

|  | **Allogeneic FMT**  **(estimate [95% CI], *p-*value)** | **Treated with D01**  **(estimate [95% CI], *p-*value)** | **Treated with D08**  **(estimate [95% CI], *p-*value)** |
| --- | --- | --- | --- |
| **HOMA-B** | estimate -15.25 [-60.65-30.39], *p-*value 0.53 | estimate 12.08 [-32.04-56.04], *p-*value 0.62 | estimate -44.42 [-88.58--0.43], *p-*value 0.09 |
| **Triglycerides (mmol/l)** | estimate -0.46 [-0.92-0.01], *p-*value 0.08 | estimate -0.61 [-1.15--0.08], ***p-*value 0.05** | estimate -0.3 [-0.84-0.24],  *p-*value 0.32 |
| **HDL cholesterol (mmol/l)** | estimate 0.02 [-0.11-0.14], *p-*value 0.81 | estimate 0.03 [-0.13-0.18],  *p-*value 0.76 | estimate 0.01 [-0.15-0.16], *p-*value 0.94 |
| **LDL cholesterol (mmol/l)** | estimate 0.02 [-0.34-0.39], *p-*value 0.91 | estimate 0.03 [-0.42-0.47],  *p-*value 0.92 | estimate 0.02 [-0.43-0.46], *p-*value 0.94 |
| **Cholesterol (mmol/l)** | estimate -0.2 [-0.57-0.17],  *p-*value 0.32 | estimate -0.25 [-0.7-0.19],  *p-*value 0.31 | estimate -0.15 [-0.6-0.3],  *p-*value 0.55 |
| **Cholesterol:HDL ratio** | estimate 0.02 [-0.32-0.36], *p-*value 0.9 | estimate -0.11 [-0.5-0.29],  *p-*value 0.63 | estimate 0.14 [-0.24-0.53], *p-*value 0.5 |
| **C-reactive protein (mg/l)** | estimate -0.31 [-0.81-0.2],  *p-*value 0.26 | estimate -0.42 [-1.04-0.19], *p-*value 0.21 | estimate -0.19 [-0.8-0.43],  *p-*value 0.58 |
| **Systolic blood pressure (mmHg)** | estimate 0.39 [-7.61-8.34], *p-*value 0.93 | estimate -0.29 [-9.97-9.45], *p-*value 0.96 | estimate 0.97 [-8.71-10.7], *p-*value 0.86 |
| **Diastolic blood pressure (mmHg)** | estimate 0.28 [-3.66-4.21], *p-*value 0.89 | estimate 0.6 [-4.04-5.25],  *p-*value 0.82 | estimate -0.11 [-4.98-4.78], *p-*value 0.97 |
| **Body mass index (kg/m2)** | estimate 0.24 [-0.35-0.83], *p-*value 0.45 | estimate 0.63 [-0.02-1.27],  *p-*value 0.09 | estimate -0.17 [-0.82-0.49], *p-*value 0.64 |
| **Abdominal circumference (cm)** | estimate -0.75 [-3.54-2.03], *p-*value 0.61 | estimate -0.4 [-3.83-3.04],  *p-*value 0.83 | estimate -1.1 [-4.47-2.28],  *p-*value 0.56 |
| **ASAT (U/l)** | estimate -1.08 [-8.03-5.86], *p-*value 0.76 | estimate 2.86 [-5.45-11.17], *p-*value 0.51 | estimate -4.59 [-12.6-3.42], *p-*value 0.28 |
| **ALAT U/l)** | estimate -3.97 [-10.3-2.35], *p-*value 0.23 | estimate -1.37 [-9.02-6.27], *p-*value 0.73 | estimate -6.55 [-14.18-1.08], *p-*value 0.1 |
| **Alkalic phosphatase (U/l)** | estimate -0.26 [-5.85-5.34], *p-*value 0.93 | estimate 2.15 [-4.68-8.97],  *p-*value 0.57 | estimate -2.54 [-9.21-4.13], *p-*value 0.5 |
| **gGT (U/l)** | estimate -2.53 [-7.67-2.6],  *p-*value 0.34 | estimate -2.5 [-8.76-3.77],  *p-*value 0.45 | estimate -2.57 [-8.83-3.68], *p-*value 0.43 |
| **bilirubin (µmol/l)** | estimate 0.13 [-1.49-1.73], *p-*value 0.88 | estimate -0.22 [-2.17-1.74], *p-*value 0.84 | estimate 0.47 [-1.5-2.44],  *p-*value 0.67 |
| **Albumin (g/l)** | estimate -0.75 [-2.02-0.52], *p-*value 0.28 | estimate -0.19 [-1.69-1.31], *p-*value 0.82 | estimate -1.31 [-2.81-0.18], *p-*value 0.12 |

FMT: faecal microbiota transplantation; D01: donor 1; D08: donor 8; HOMA-B: Homeostasis Model Assessment of pancreatic β-cell function; HDL: high-density lipoprotein; LDL: low-density lipoprotein; ASAT: aspartate aminotransferase; ALAT: alanine aminotransferase; gGT: gamma-glutamyltransferase.

**Supplementary table 2. Effects of consecutive allogeneic FMT on metabolic parameters over time**

|  |  | **Allogeneic FMT**  **(estimate [95% CI], *p-*value)** | **Treated with D01**  **(estimate [95% CI], *p-*value)** | **Treated with D08**  **(estimate [95% CI], *p-*value)** |
| --- | --- | --- | --- | --- |
| **AUC** | **Glucose (mmol/l)*minutes** | -8.64 [-151.65-134.36],  *p-*value 0.9 | 4.78 [-166.88-176.45],  *p-*value 0.96 | -22.69 [-205.41-160.03],  *p-*value 0.8 |
|  | **Insulin (mIU/l)*minutes** | -33.61 [-4012.35-3945.12],  *p-*value 0.99 | 671.95 [-4200.8-5544.7],  *p-*value 0.78 | -862.23 [-5984.32-4259.86],  *p-*value 0.74 |
|  | **C-peptide (nmol/l)*minutes** | 13.06 [-42.32-68.45],  *p-*value 0.64 | 21.97 [-47.18-91.12],  *p-*value 0.53 | 4.05 [-65.92-74.03],  *p-*value 0.91 |
|  | **GIP (pmol/l)*minutes** | -7.33 [-2270.59-2257.37],  *p-*value 1 | 246.18 [-2500.95-2991.59],  *p-*value 0.87 | -261.22 [-3019.72-2495.69],  *p-*value 0.86 |
|  | **GLP*-*1 (pmol/l)*minutes** | -127.32 [-426.31-172.94],  *p-*value 0.43 | -109.35 [-468.89-248.85],  *p-*value 0.59 | -143.41 [-509.85-221.19],  *p-*value 0.48 |
| **iAUC** | **Glucose (mmol/l)*minutes** | -14.07 [-115.14-87],  *p-*value 0.78 | 4.47 [-113.2-122.14],  *p-*value 0.94 | -32.68 [-158.9-93.53],  *p-*value 0.6 |
|  | **Insulin (mIU/l)*minutes** | 299.66 [-3657.04-4256.37], *p-*value 0.88 | 432.61 [-4388.01-5253.22], *p-*value 0.86 | 99.04 [-5031.05-5229.14],  *p-*value 0.97 |
|  | **C-peptide (nmol/l)*minutes** | 27.72 [-29.75-85.2],  *p-*value 0.34 | 25.69 [-44.67-96.05],  *p-*value 0.47 | 30.1 [-44.28-104.48],  *p-*value 0.42 |
|  | **GIP (pmol/l)*minutes** | 150.55 [-2285.59-2588.23], *p-*value 0.91 | 506.83 [-2426.48-3438.37], *p-*value 0.76 | -217.8 [-3190.44-2753.14], *p-*value 0.89 |
|  | **GLP-1 (pmol/l)*minutes** | 29.42 [-343.41-402.7],  *p-*value 0.88 | 78.98 [-371.06-528.53],  *p-*value 0.75 | -19.3 [-469.33-430.24],  *p-*value 0.94 |

FMT: faecal microbiota transplantation; D01: donor 1; D08: donor 8; GIP: Gastric inhibitory polypeptide; GLP-1: Glucagon-like peptide-1.

| **Donor** | **Responders W3*** | **Responders W6*** | **Responders W12*** |
| --- | --- | --- | --- |
| **D01** | 0 | **2** | **2** |
| **D08** | 0 | **1** | **2** |
| **Autologous FMT** | 0 | **3** | **3** |

**Supplemental table 3 Responders**

*Response defined as >5% decrease in MRI-PDFF, HOMA-IR and serum triglycerides. In case of missing values, response was based on combined decrease in MRI-PDFF and triglycerides (6/20). FMT: faecal microbiota transplantation; D01: donor 1; D08: donor 8.

**Supplementary table 4 Metabolic parameters versus response/non-response**

|  |  | **Response (total)**  **(estimate [95% CI], *p*-value)** | **Response (allogeneic FMT)**  **(estimate [95% CI], *p*-value)** |
| --- | --- | --- | --- |
| **AUC** | **Glucose (mmol/l)*minutes** | -107.92 [-241.14-25.3],  *p-*value 0.11 | -18.94 [-149.93-112.04],  *p-*value 0.77 |
|  | **Insulin (mIU/l)*minutes** | -3016.46 [-6839.04-806.11],  *p-*value 0.12 | -4894.43 [-9303.56--485.29],  ***p-*value 0.03*** |
|  | **C-peptide (nmol/l)*minutes** | -59.14 [-109.46--8.81],  ***p-*value 0.02*** | -56.67 [-114.54-1.2],  *p-*value 0.05 |
|  | **GIP (pmol/l)*minutes** | 444.09 [-1945.05-2830.91],  *p-*value 0.73 | -476.6 [-3673.46-2720.25],  *p-*value 0.79 |
|  | **GLP-1 (pmol/l)*minutes** | -126.88 [-447.07-192.23],  *p-*value 0.46 | 25.24 [-294.35-344.82],  *p-*value 0.89 |
| **iAUC** | **Glucose (mmol/l)*minutes** | -52.88 [-150.43-44.68],  *p-*value 0.28 | 3.64 [-89.48-96.76],  *p-*value 0.94 |
|  | **Insulin (mIU/l)*minutes** | -2700.47 [-6565.61-1164.67],  *p-*value 0.17 | -3309.51 [-8027.74-1408.72],  *p-*value 0.16 |
|  | **C-peptide (nmol/l)*minutes** | -46.53 [-103.89-10.83],  *p-*value 0.11 | -15.24 [-79.29-48.81],  *p-*value 0.63 |
|  | **GIP (pmol/l)*minutes** | 398.04 [-2184.34-2977.92],  *p-*value 0.77 | -308.15 [-3834.79-3218.49],  *p-*value 0.88 |
|  | **GLP-1 (pmol/l)*minutes** | 0.46 [-401.5-401.71],  *p-*value 1 | -46.33 [-501.38-408.73],  *p-*value 0.86 |

**p-*values <0.05 were depicted in bold. FMT: faecal microbiota transplantation; GIP: Gastric inhibitory polypeptide; GLP-1: Glucagon-like peptide-1.

**Supplementary table 5 Association of microbial diversity with MRI-PDFF, HOMA-IR and serum triglycerides over time and by treatment group.**

|  | **Irrespective of treatment**  **(estimate [95% CI], *p*-value)** | **Allogeneic versus autologous FMT**  **(estimate [95% CI], *p*-value)** |
| --- | --- | --- |
| **ΔMRI-PDFF** | -0.02 [-0.06-0.01], *p*-value 0.24 | 0.13 [0.06-0.19], ***p*-value 0.002*** |
| **ΔHOMA-IR** | 0.01 [-0.01-0.03], *p*-value 0.52 | -0.03 [-0.07-0.01], *p*-value 0.17 |
| **ΔTriglycerides** | 0.02 [-0.11-0.15], *p*-value 0.75 | 0.03 [-0.28-0.32], *p*-value 0.88 |

**p-*values <0.05 were depicted in bold. Linear mixed models were used to assess the association between microbiota diversity (Shannon) and potential markers of clinical response to treatment. Models were fit both irrespective of treatment and with an interaction term to assess whether the relationship between Shannon diversity and clinical markers differed between treatment groups (Supplement 1). FMT: faecal microbiota transplantation; MRI-PDFF: magnetic resonance imaging-derived proton density fat fraction; HOMA-IR: homeostasis model assessment of insulin resistance.

**Supplementary table 6. Longitudinal differential abundance of top 35 taxa between allogeneic and autologous FMT groups.**

|  |  | **Log2 fold change** | **SE** | **Adjusted *p-*value** |
| --- | --- | --- | --- | --- |
| **Family** | *Rhodocyclaceae* | 1.60 | 0.41 | 0.08 |
| **Genus** | *-* | - | - | - |
| **Species** | *Prevotella sp900557255* | 6.63 | 1.47 | 0.08 |
|  | *Prevotella copri* | 5.25 | 1.28 | 0.08 |
|  | *Prevotella sp002297965* | 5.14 | 1.17 | 0.08 |
|  | *Prevotella sp900544825* | 5.03 | 1.24 | 0.08 |
|  | *Prevotella sp900557035* | 4.90 | 1.32 | 0.10 |
|  | *Prevotella sp900314195* | 4.28 | 1.00 | 0.08 |
|  | *Alcanivorax profundi* | 4.26 | 1.33 | 0.19 |
|  | *Prevotella sp900551275* | 4.23 | 1.01 | 0.08 |
|  | *Prevotella sp900551985* | 4.22 | 1.44 | 0.23 |
|  | *Prevotella sp900555035* | 3.95 | 0.97 | 0.08 |
|  | *Prevotella sp900548535* | 3.73 | 1.04 | 0.12 |
|  | *Prevotella copri_A* | 3.62 | 1.18 | 0.19 |
|  | *Prevotella sp900546535* | 3.43 | 0.89 | 0.10 |
|  | *Prevotella hominis* | 3.10 | 1.04 | 0.21 |
|  | *Prevotella sp002251295* | 3.08 | 0.77 | 0.08 |
|  | *Prevotella sp900556795* | 3.03 | 0.95 | 0.19 |
|  | *Prevotella sp002440225* | 2.80 | 0.91 | 0.19 |
|  | *Prevotella sp900554835* | 2.72 | 0.78 | 0.14 |
|  | *Catenibacterium sp900540685* | 2.65 | 0.70 | 0.19 |
|  | *Clostridium sp000435835* | 2.65 | 0.98 | 0.27 |
|  | *Senegalimassilia faecalis* | 2.49 | 0.77 | 0.17 |
|  | *Terrisporobacter sp900557165* | 2.11 | 0.67 | 0.19 |
|  | *Holdemanella sp900547815* | 2.05 | 0.56 | 0.08 |
|  | *Prevotella sp900770515* | 2.02 | 0.62 | 0.14 |
|  | *Prevotella sp900767615* | 1.69 | 0.55 | 0.19 |
|  | *Gemmiger formicilis* | 1.48 | 0.51 | 0.19 |
|  | *Holdemanella sp002299315* | 1.47 | 0.53 | 0.21 |
|  | *Prevotella sp900556825* | 1.26 | 0.34 | 0.12 |
|  | *Holdemanella biformis* | 1.07 | 0.40 | 0.27 |
|  | *Enterocloster sp900543885* | -1.00 | 0.35 | 0.23 |
|  | *Bacteroides sp900552405* | -1.18 | 0.39 | 0.19 |
|  | *Bacteroides acidifaciens_A* | -1.29 | 0.44 | 0.19 |
|  | *Bacteroides intestinalis_A* | -1.43 | 0.49 | 0.19 |
|  | *Akkermansia muciniphila* | -4.91 | 1.29 | 0.10 |

Top 35 taxa were selected based on adjusted *p-*values (Benjamini-Hochberg). Linear mixed models (Supplement 1) are described in Supplement 1. FMT: faecal microbiota transplantation; SE: standard error of log2 fold change.

**Supplementary table 7. Longitudinal differential abundance of top 35 taxa associated with response status, irrespective of FMT treatment group.**

|  |  | **Log2 fold change** | **SE** | **Adjusted *p-*value** |
| --- | --- | --- | --- | --- |
| **Family** | *UBA1067* | 1.64 | 0.82 | 0.81 |
|  | *Alcanivoracaceae* | 1.48 | 1.43 | 0.85 |
|  | *CAG-138* | 1.44 | 0.59 | 0.81 |
|  | *UBA644* | 1.11 | 0.90 | 0.81 |
|  | *Borkfalkiaceae* | 1.07 | 0.62 | 0.81 |
|  | *UBA660* | 0.95 | 0.64 | 0.81 |
|  | *Desulfovibrionaceae* | 0.89 | 0.64 | 0.81 |
|  | *Acidaminococcaceae* | 0.83 | 0.54 | 0.81 |
|  | *Chitinophagaceae* | 0.79 | 0.43 | 0.81 |
|  | *QAND01* | 0.78 | 0.45 | 0.81 |
|  | *Anaerotignaceae* | 0.72 | 0.34 | 0.81 |
|  | *Erysipelotrichaceae* | 0.69 | 0.31 | 0.81 |
|  | *CAG-508* | 0.67 | 0.67 | 0.85 |
|  | *CAG-272* | 0.60 | 0.48 | 0.81 |
|  | *CAG-74* | 0.59 | 0.47 | 0.81 |
|  | *Peptococcaceae* | 0.57 | 0.32 | 0.81 |
|  | *CAG-977* | 0.57 | 0.49 | 0.81 |
|  | *Erysipelatoclostridiaceae* | 0.54 | 0.43 | 0.81 |
|  | *Paludibacteraceae* | 0.53 | 0.38 | 0.81 |
|  | *Atopobiaceae* | 0.43 | 0.61 | 0.86 |
|  | *Muribaculaceae* | 0.43 | 0.36 | 0.81 |
|  | *Acutalibacteraceae* | 0.41 | 0.26 | 0.81 |
|  | *DTU023* | 0.36 | 0.32 | 0.82 |
|  | *Oscillospiraceae* | 0.34 | 0.29 | 0.81 |
|  | *Bacteroidaceae* | 0.33 | 0.37 | 0.86 |
|  | *Eggerthellaceae* | 0.26 | 0.29 | 0.86 |
|  | *Butyricicoccaceae* | 0.23 | 0.23 | 0.85 |
|  | *P3* | -0.19 | 0.33 | 0.86 |
|  | *Lachnospiraceae* | -0.26 | 0.19 | 0.81 |
|  | *Coprobacteraceae* | -0.27 | 0.35 | 0.86 |
|  | *Bifidobacteriaceae* | -0.33 | 0.54 | 0.86 |
|  | *CAG-288* | -0.88 | 0.83 | 0.85 |
|  | *UBA11471* | -1.08 | 0.95 | 0.82 |
|  | *Akkermansiaceae* | -1.21 | 1.02 | 0.81 |
|  | *UBA5633* | -1.79 | 1.35 | 0.81 |
| **Genus** | *-* | - | - | - |
| **Species** | *-* | - | - | - |

Top 35 taxa were selected based on adjusted *p-*values (Benjamini-Hochberg). Linear mixed models (Supplement 1) are described in Supplement 1. FMT: faecal microbiota transplantation; SE: standard error of log2 fold change.

**Supplementary table 8. Longitudinal differential abundance of the top 35 taxa associated with response status, compared between allogeneic and autologous FMT.**

|  |  | **log2 fold change** | **SE** | **Adjusted *p-*value** |
| --- | --- | --- | --- | --- |
| **Family** | *Fusobacteriaceae* | 2.06 | 1.97 | 0.80 |
|  | *UBA1820* | 2.00 | 2.08 | 0.80 |
|  | *Barnesiellaceae* | 1.58 | 1.38 | 0.80 |
|  | *CAG-449* | 1.30 | 0.91 | 0.80 |
|  | *CAG-313* | 1.17 | 1.03 | 0.80 |
|  | *CAG-274* | 0.92 | 1.03 | 0.80 |
|  | *Tannerellaceae* | 0.71 | 0.93 | 0.80 |
|  | *P3* | 0.70 | 0.67 | 0.80 |
|  | *Chitinophagaceae* | 0.69 | 0.88 | 0.80 |
|  | *Coprobacteraceae* | 0.69 | 0.72 | 0.80 |
|  | *Veillonellaceae* | 0.66 | 0.75 | 0.80 |
|  | *Bacteroidaceae* | 0.62 | 0.76 | 0.80 |
|  | *Butyricicoccaceae* | 0.50 | 0.46 | 0.80 |
|  | *Ruminococcaceae* | -0.38 | 0.39 | 0.80 |
|  | *Oscillospiraceae* | -0.62 | 0.60 | 0.80 |
|  | *CAG-272* | -0.77 | 1.01 | 0.80 |
|  | *Coriobacteriaceae* | -0.78 | 0.75 | 0.80 |
|  | *Paludibacteraceae* | -0.78 | 0.80 | 0.80 |
|  | *CAG-74* | -0.81 | 0.97 | 0.80 |
|  | *Eggerthellaceae* | -0.82 | 0.57 | 0.80 |
|  | *DTU023* | -0.86 | 0.66 | 0.80 |
|  | *CAG-826* | -0.90 | 1.09 | 0.80 |
|  | *Peptococcaceae* | -0.92 | 0.65 | 0.80 |
|  | *Bifidobacteriaceae* | -1.01 | 1.14 | 0.80 |
|  | *CAG-508* | -1.15 | 1.36 | 0.80 |
|  | *Selenomonadaceae* | -1.15 | 1.25 | 0.80 |
|  | *Megasphaeraceae* | -1.17 | 1.02 | 0.80 |
|  | *Gastranaerophilaceae* | -1.22 | 1.11 | 0.80 |
|  | *Lactobacillaceae* | -1.22 | 1.08 | 0.80 |
|  | *Muribaculaceae* | -1.44 | 0.66 | 0.80 |
|  | *Streptococcaceae* | -1.48 | 1.42 | 0.80 |
|  | *UBA644* | -1.52 | 1.86 | 0.80 |
|  | *Atopobiaceae* | -1.64 | 1.13 | 0.80 |
|  | *QAMH01* | -2.22 | 1.64 | 0.80 |
|  | *Methanobacteriaceae* | -2.36 | 1.22 | 0.80 |
| **Genus** | *-* | - | - | - |
| **Species** | *-* | - | - | - |

Top 35 taxa were selected based on adjusted *p-*values (Benjamini-Hochberg). Linear mixed models (Supplement 1) are described in Supplement 1. FMT: faecal microbiota transplantation; SE: standard error of log2 fold change.

**Supplementary table 9 Longitudinal differential abundance of the top 35 taxa associated with individual response parameters, compared between allogeneic and autologous FMT.**

|  |  |  | **Log2 fold**  **change** | **SE** | **Adjusted *p-*value** |
| --- | --- | --- | --- | --- | --- |
| **HOMA-IR** | **Family** | *Ruminococcaceae* | 0.26 | 0.09 | 0.37 |
|  | **Genus** | *51-20* | 1.87 | 0.55 | 0.43 |
|  |  | *Frisingicoccus* | 0.71 | 0.19 | 0.17 |
|  | **Species** | *Dialister invisus* | 2.29 | 0.59 | 0.31 |
| **Serum triglycerides** | **Family** | *Alcanivoracaceae* | 1.88 | 0.56 | 0.12 |
|  |  | *Methanobacteriaceae* | 0.88 | 0.30 | 0.19 |
|  |  | *CAG-382* | 0.50 | 0.21 | 0.43 |
|  |  | *Coriobacteriaceae* | 0.44 | 0.18 | 0.43 |
|  | **Genus** | *Alcanivorax* | 1.73 | 0.58 | 0.39 |
|  |  | *CAG-267* | 1.42 | 0.59 | 0.44 |
|  |  | *Methanobrevibacter_A* | 0.98 | 0.31 | 0.30 |
|  |  | *UBA1394* | 0.92 | 0.39 | 0.44 |
|  |  | *Ellagibacter* | 0.85 | 0.32 | 0.44 |
|  |  | *UBA11774* | 0.74 | 0.28 | 0.44 |
|  |  | *Zag1* | 0.69 | 0.18 | **0.05*** |
|  |  | *Ruminococcus_F* | 0.60 | 0.25 | 0.44 |
|  |  | *Collinsella* | 0.45 | 0.19 | 0.44 |
|  |  | *Frisingicoccus* | 0.44 | 0.18 | 0.44 |
|  |  | *GCA-900066135* | 0.36 | 0.14 | 0.44 |
|  |  | *Hydrogenoanaerobacterium* | 0.28 | 0.11 | 0.44 |
|  |  | *TF01-11* | -0.68 | 0.28 | 0.44 |
|  |  | *UBA940* | -0.90 | 0.22 | 0.05 |
|  | **Species** | *Alcanivorax profundi* | 2.22 | 0.54 | 0.07 |
|  |  | *Prevotella sp900557035* | 2.15 | 0.54 | 0.07 |
|  |  | *Prevotella copri* | 2.08 | 0.51 | 0.07 |
|  |  | *Prevotella sp002297965* | 1.56 | 0.51 | 0.33 |
|  |  | *Prevotella sp900544825* | 1.49 | 0.50 | 0.33 |
|  |  | *Prevotella sp900551275* | 1.49 | 0.42 | 0.16 |
|  |  | *Methanobrevibacter_A sp900766745* | 1.21 | 0.32 | 0.07 |
|  |  | *UMGS882 sp900757905* | 1.14 | 0.30 | 0.07 |
|  |  | *Catenibacterium sp900540685* | 1.04 | 0.35 | 0.33 |
|  |  | *Catenibacterium sp900764725* | 0.79 | 0.24 | 0.29 |
|  |  | *UBA7160 sp902363665* | 0.43 | 0.14 | 0.32 |
|  |  | *Schaedlerella sp004556565* | 0.30 | 0.09 | 0.29 |
|  |  | *UBA940 sp900768115* | -1.25 | 0.25 | **0.02*** |
| **MRI-PDFF** | **Family** | - | - | - | - |
|  | **Genus** | - | - | - | - |
|  | **Species** | - | - | - | - |

*Adjusted *p-*values <0.05 were depicted in bold. Top 35 taxa were selected based on adjusted *p-*values (Benjamini-Hochberg). Linear mixed models (Supplement 1) are described in Supplement 1. FMT: faecal microbiota transplantation; SE: standard error of log2 fold change.

**Supplementary table 10 Association of species engraftment fraction with MRI-PDFF, HOMA-IR and serum triglycerides in patients treated with allogeneic FMT**

|  | **Treated with allogeneic FMT (estimate [95% CI], *p*-value)** |
| --- | --- |
| **ΔMRI-PDFF** | -0.52 [-2.15-1.11], *p-*value 0.61 |
| **ΔHOMA-IR** | -0.41 [-1.14-0.54], *p-*value 0.31 |
| **ΔTriglycerides** | -2.75 [-11.03-5.4], *p-*value 0.52 |

Linear mixed models were used to assess the association between donor species engraftment in the patient and potential markers of clinical response to treatment (Supplement 1). FMT: faecal microbiota transplantation; MRI-PDFF: magnetic resonance imaging-derived proton density fat fraction; HOMA-IR: homeostasis model assessment of insulin resistance.

**Supplementary table 11 Association of patient microbiota similarity with MRI-PDFF, HOMA-IR and serum triglycerides in patients treated with allogeneic FMT.**

|  | **Treated with allogeneic FMT (estimate [95% CI], *p*-value)** |
| --- | --- |
| **MRI-PDFF** | -0.01 [-0.02-0], *p-*value 0.1 |
| **HOMA-IR** | 0 [-0.01-0.02], *p-*value 0.5 |
| **Triglycerides** | -0.07 [-0.14-0.09], *p-*value 0.33 |

Linear mixed models were used to assess the association between patient microbiota similarity to the donor and potential markers of clinical response to treatment (Supplement 1). FMT: faecal microbiota transplantation; MRI-PDFF: magnetic resonance imaging-derived proton density fat fraction; HOMA-IR: homeostasis model assessment of insulin resistance.
